# Visualizing Metabolism: Lipid-Based Raman Nanoreporters as an Enabling Platform for Metabolic Imaging and Precision Medicine

**DOI:** 10.1021/acsnanomed.6c00039

**Published:** 2026-09-22

**Authors:** Anu Yadav, Sreejita Pal, Arghyadeep Mayur, Joysmita Dutta, Suresh K. Verma, Manas Ranjan Gartia

**Affiliations:** † Department of Mechanical and Industrial Engineering, 5779 Louisiana State University, Baton Rouge, Louisiana 70803, United States; ‡ School of Biotechnology, Kalinga Institute of Industrial Technology, Bhubaneswar, Odisha 751024, India

**Keywords:** Raman spectroscopy, Metabolic imaging, Lipid nanoparticles, Bioorthogonal Raman imaging, Stable isotope probing (SIP), SRS, SERS, Metabolic reprogramming

## Abstract

Lipid-based Raman reporters represent a promising but still developing class of metabolic sensors. This review reports lipid nanoparticles and lipid conjugated inorganic nanoparticles as Raman-active systems capable of decoding cellular metabolism with spatial and chemical specificity. It is demonstrated how bioorthogonal reporters operating in the Raman-silent window were incorporated across various modalities such as spontaneous Raman, coherent anti-Stokes Raman spectroscopy, stimulated Raman scattering, surface enhanced Raman spectroscopy, spatially offset Raman spectroscopy, and tip-enhanced Raman spectroscopy. Furthermore, strategies are presented for achieving multiplexed and barcoded Raman imaging using isotopic encoding and other molecular tags. The hybrid integration of Raman with other imaging modulation such as photoacoustic, magnetic resonance imaging, and positron emission tomography is evaluated to overcome inherent limitations in penetration depth and sensitivity. Additionally, the application of Raman reporters is characterized into *in vitro* and *in vivo* studies, detailing both oncological and nononcological metabolic imaging. Finally, this review outlines pathways for technical standardization, discusses challenges in regulatory approval, and provides a forward-looking perspective on the clinical translation of Raman-based metabolic nanoprobes.

## Graphical abstract

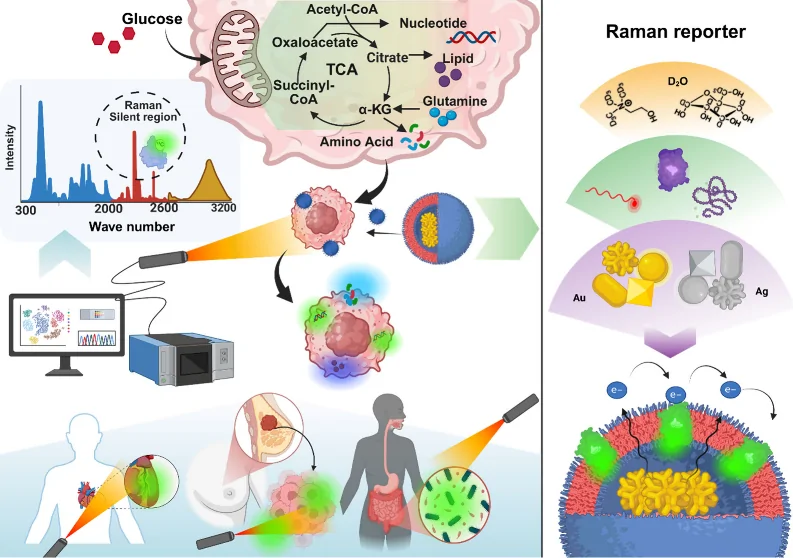

Metabolic reprogramming is a fundamental hallmark of cancer and other diseases such as neurological, metabolic, and infectious diseases. It allows the cells to sustain proliferation even under low-nutrient conditions, evade the immune system, and drive invasion and metastasis.^1,2^ Cancer cells dynamically reprogram lipid metabolism with spatial and temporal heterogeneity,^3–6^ making imaging tools that map these processes highly valuable. Within the metabolic landscape, lipid reprogramming is crucial for membrane biosynthesis, energy storage, organelle remodeling, and signaling that drive tumor progression.^7,8^ Cancer cells enhance de novo lipogenesis, accumulate lipid droplets, modify fatty acid saturation, and remodel phospholipids to support cell proliferation and adapt to stress from nutrients and cancer therapy.^9^ Conventional approaches such as wet chemical assays (e.g., Seahorse assay) and mass spectroscopy (LC-MS/MS, MALDI) remain standard for metabolic analysis, but involves homogenization of the tissue. However, there is growing interest in optical spectroscopic methods capable of capturing biochemical compositions directly within living systems.^10^ As in traditional LC-MS, this destruction of the physical tissue architecture prevents researchers from mapping metabolic heterogeneity across distinct microenvironmental niches.

Metabolism is a complex network of biochemical reactions that transforms nutrients into energy and the molecular components required for cell survival, growth, and normal function.^11^ Major pathways, including glucose, glutamine, and lipid metabolism, generate intermediates needed for nucleotide, amino acid, protein, and membrane components. These pathways also support the tricarboxylic acid (TCA) cycle and oxidative phosphorylation for adenosine triphosphate (ATP) production. Figure 1 summarizes the major metabolic pathways that support energy production and biosynthesis.^12–14^ Lipid metabolism is closely connected to these processes. It provides fatty acids for membrane formation, signaling, and energy generation through β-oxidation. The synthesis of fatty acids and cellular membranes depends on both carbon precursors and nicotinamide adenine dinucleotide phosphate (NADPH). As a result, lipid biogenesis relies strongly on glucose-derived metabolic flux.^15^ Because these pathways are highly dynamic and interconnected, imaging technologies are essential for studying metabolism at the tissue, cellular, and organelle levels.

**Figure 1:**
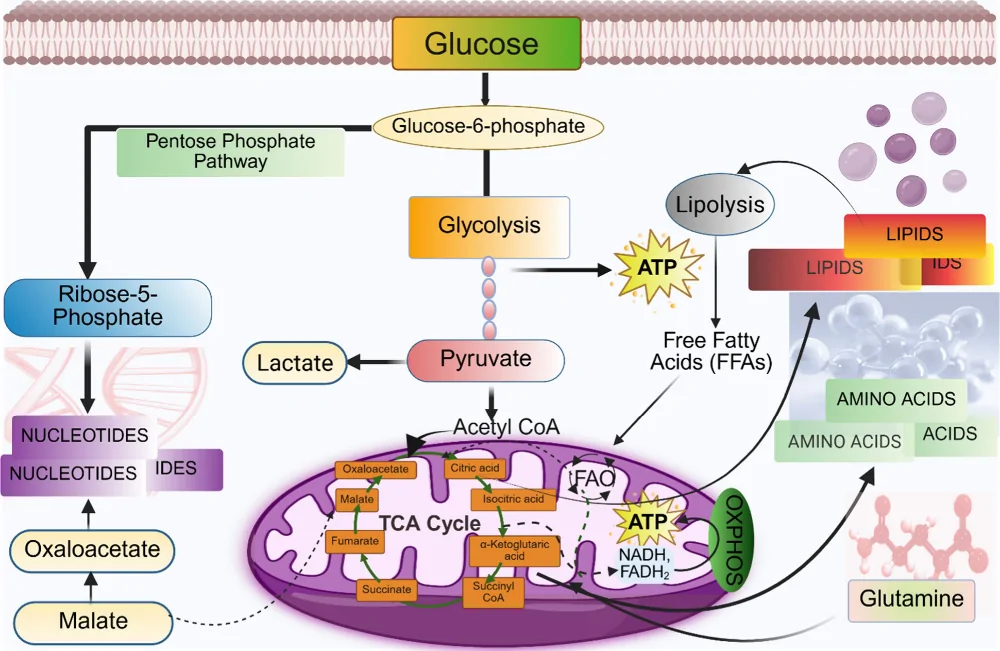
Schematic representation of major metabolic processes in a cell to produce energy. Glucose is converted to glucose-6-phosphate and then enters the glycolysis pathway, generating pyruvate, ATP, and lactate. Pyruvate is converted to acetyl-CoA and feeds the tricarboxylic acid (TCA) cycle and produces intermediates that support biosynthesis and oxidative phosphorylation. Carbon from glucose is also routed through pentose phosphate pathway to generate ribose-5-phosphate for nucleotide synthesis. TCA cycle intermediates and related metabolites contribute to the synthesis of lipids, amino acids, and nucleotides. Free fatty acids in the cell cytoplasm enter mitochondria to undergo fatty acid oxidation (FAO) to produce acetyl-CoA, NADH, and ATP. The image is created using BioRender.com.

Whole-body imaging approaches such as positron emission tomography (PET),^16^ magnetic resonance imaging (MRI), and magnetic resonance spectroscopy (MRS) provide metabolic and anatomical information.^17,18^ PET, CT, and MRI/MRS are highly biocompatible, noninvasive imaging methods. They still lack subcellular resolution with typical resolution ∼ 1 mm^19^ and have limited sensitivity and metabolic specificity.^20^ They use radiotracers like fluoro deoxyglucose (FDG) to assess glucose uptake or require exogenous contrast agents that may induce toxicity or expose tissues to ionizing radiation.^21^ Major progress has been achieved using liquid chromatography–mass spectrometry (LC-MS), an established gold standard. It enables sensitive, untargeted/targeted metabolite profiling.^22^ Bulk lipidomics using LC-MS offers high-resolution molecular profiling.^23^ However, LC-MS relies on sample homogenization, resulting in loss of spatial context.^24^ For example, in a tissue, signals from epithelial, stromal, and immune regions would be combined into an average profile. Although LC-MS can reveal lipid enrichment, it cannot determine where these metabolic alterations arise within the tumor microenvironment. Spatially resolved metabolic analysis is therefore critical, as compartment-specific lipid states can influence tumor progression, treatment resistance, and therapeutic vulnerability.^11^ Mass spectrometry imaging (MSI) partially overcomes this limitation by localizing lipid, protein and metabolite distributions within heterogeneous tumors without prior labeling.^25,24^ Nevertheless, MSI still requires demanding sample preparation, including cryosectioning, matrix deposition, vacuum compatibility for matrix-assisted laser desorption/ionization (MALDI)-based methods, and extensive postacquisition normalization. Samples are also typically mounted on conductive indium tin oxide (ITO) slides for analysis. In addition, MSI platforms are expensive and often have limited throughput, which can restrict large-scale or rapid studies.^26,27^ Unlike MS, nuclear magnetic resonance (NMR) spectroscopy provides inherently quantitative measurements without extensive sample preparation, although its sensitivity remains lower than MS.^28^ These limitations create a need for complementary imaging approaches that can provide chemical specificity while preserving spatial context in living systems.

Among optical imaging techniques, fluorescence microscopy offers highly sensitive and real-time visualization of metabolites on the subcellular scale. However, fluorescence imaging depends on attaching fluorescent labels that are substantially larger than the metabolites being studied.^20,29^ They may alter natural metabolite behavior, and currently remain limited to relatively few metabolic targets.^30^ Since the 1950s, endogenous NAD­(P)H and flavin adenine dinucleotide (FAD) autofluorescence imaging has been widely used to study metabolic states in tissue and living cells.^31^ The metabolic activities can be studied using endogenous fluorescence imaging of NAD­(P)H and FAD, which reflects the cellular metabolic state. Measuring the fluorescence signals of these cofactors can help estimate cellular metabolic activity, because metabolic potential is closely related to the balance between reduced and oxidized metabolic substrates.^32^ Fluorescence lifetime imaging microscopy (FLIM) further enables quantification of free and protein-bound NAD­(P)­H, allowing assessment of shifts between glycolysis and oxidative phosphorylation.^31^ Fluorescent probes can also detect ATP, reactive oxygen species, glucose uptake, membrane potential, and pH, although labels may disturb natural metabolism and often limit multiplexed analysis. Research has shown that the optical redox ratio (FAD/(NADH+FAD)) and NADH lifetime can distinguish different cancer cell phenotypes and metabolic responses.^21^ Recently, multiphoton fluorescence lifetime imaging microscopy (MPF-FLIM) has been widely used for high-resolution, label-free monitoring of metabolic changes in living tissues and cells. Although these techniques are rapid and nondestructive, shallow imaging depth, high instrument cost, and complex data analysis still limit broader application.^31^ Moreover, spectral crosstalk and interference from autofluorescent molecules such as keratin, elastin, melanin, and lipofuscin can affect accuracy.^33^ Additional optical approaches such as second harmonic generation (SHG) and photoacoustic imaging provide complementary structural and functional information.^34,35^ However, they do not directly measure metabolism with high molecular specificity.^36–38^ Therefore, there is a strong need for techniques capable of quantitatively mapping TCA-associated metabolic activity at the subcellular level with label-free approaches.

To better understand the cellular metabolism of biomolecules such as lipids, glucose, and proteins, imaging methods with high spatial resolution and chemical specificity are essential. Among the available techniques, vibrational microscopy has emerged as an important complementary imaging tool. It detects chemical bonds directly with a high specificity and minimal labeling requirements. Raman microscopy, based on inelastic scattering, provides label-free chemical imaging via vibrational fingerprints. However, spontaneous Raman imaging is limited by weak signals and slow acquisition.^21^ Surface-enhanced Raman spectroscopy (SERS) arises from electromagnetic hotspots that amplify Raman signals by orders of magnitude, enabling single-molecule detection.^39,40^ Coherent anti-Stokes Raman (CARS) and stimulated Raman scattering (SRS) further advanced label-free lipid imaging and live-cell metabolic analysis.^41^ Early SRS studies enabled high-throughput imaging of lipid droplet dynamics and identification of fat-regulatory genes without conventional staining.^42^

Although label-free vibrational microscopy can reveal the native chemical landscape of biological systems, it can also overlap with signals within the fingerprint region. In cellular Raman spectra, most endogenous signals appear either in the fingerprint region (400–1800 cm^–1^) or in the C–H stretching region (2700–3200 cm^–1^). Therefore, there exists a relatively signal-free “silent region” (1800–2700 cm^–1^).^43^ Further, the weak-signal nature of Raman spectroscopy hinders the selective resolution of specific metabolic processes, making it challenging to detect low-abundance metabolites, pathway-specific intermediates, and chemically similar species. To address these limitations, vibrational probes, Raman tags, and stable isotope labeling approaches have been developed. These strategies enhance sensitivity, molecular selectivity, and multiplexing capability. Over the past decade, several Raman-active probes based on carbon triple bonds have gained prominence. These probes produce distinct signals in the silent region, enabling background-free and photobleaching-resistant detection.^44^ Common functional groups include alkynes (C≡C), nitriles (C≡ N), and deuterated carbon (C–D), which are small, minimally polar, and less perturbative compared to conventional fluorescent labels.^45^ Among these, alkyne tags are particularly attractive due to their small size, low endogenous abundance, and chemical inertness toward native biomolecules. Compared with carbon-deuterium (C–D) labels, alkynes generate substantially stronger Raman signals, making them highly effective for dynamic live -cell imaging.^46^

Using these compact vibrational tags, numerous small biomolecules—including nucleosides, fatty acids, cholesterol, amino acids, choline, and glucose—have been successfully imaged in living cells and tissues.^47^ In lipid biology, deuterium- or alkyne-tagged probes have successfully mapped fatty acid uptake, storage, and trafficking pathways that promote endoplasmic reticulum (ER) stress or lipid peroxidation during ferroptosis. Beyond lipids, vibrational probes are widely applied to trace glucose metabolism. Alkyne-tagged glucose derivatives allow researchers to quantify cellular glucose uptake,^48^ while deuterated glucose tracers can be tracked as they are incorporated into downstream lipids,^49^ proteins, nucleic acids,^50^ and glycogen. Furthermore, deuterium oxide (D_2_O) labeling enables global imaging of newly synthesized lipids and other metabolic turnover rates *in vivo*.^51^

Despite these capabilities, a significant translational hurdle remains. Free small-molecule probes generally require protective encapsulation to maintain structural stability, prevent rapid enzymatic degradation, and ensure optimal signal delivery within complex cellular environments.^52^ To address these delivery limitations, researchers have increasingly turned to the principles of nanomedicine^53,54^ (Figure 2). Over the past few decades, engineering nanoscale therapeutics has gained tremendous momentum to improve efficacy in solid tumors, target specific tissue niches, and enhance systemic biodistribution.^55^ A vast array of organic and inorganic materials has been evaluated for nanoparticle (NP) fabrication, including polymers, metallic nanostructures,^56^ hydrogels,^57,58^ nanocrystals, carbon nanostructures,^59,60^ and dendrimers,^18^ for both diagnostic and therapeutic purposes. These systems can pack bioactive cargo with high density, improving bioavailability across diverse disease models.^61,62^ An early paradigm for using this in vibrational imaging was demonstrated by Min and co-workers, who embedded alkyne-based reporters in engineered polystyrene nanodots. By doping these nanodots with alkyne, nitrile, and C–D tags, they generated distinct, nonoverlapping Raman peaks at 2163, 2232, and 2293 cm^–1^, facilitating background-free, three-color multiplexed imaging in live cells.^63,64^

**Figure 2:**
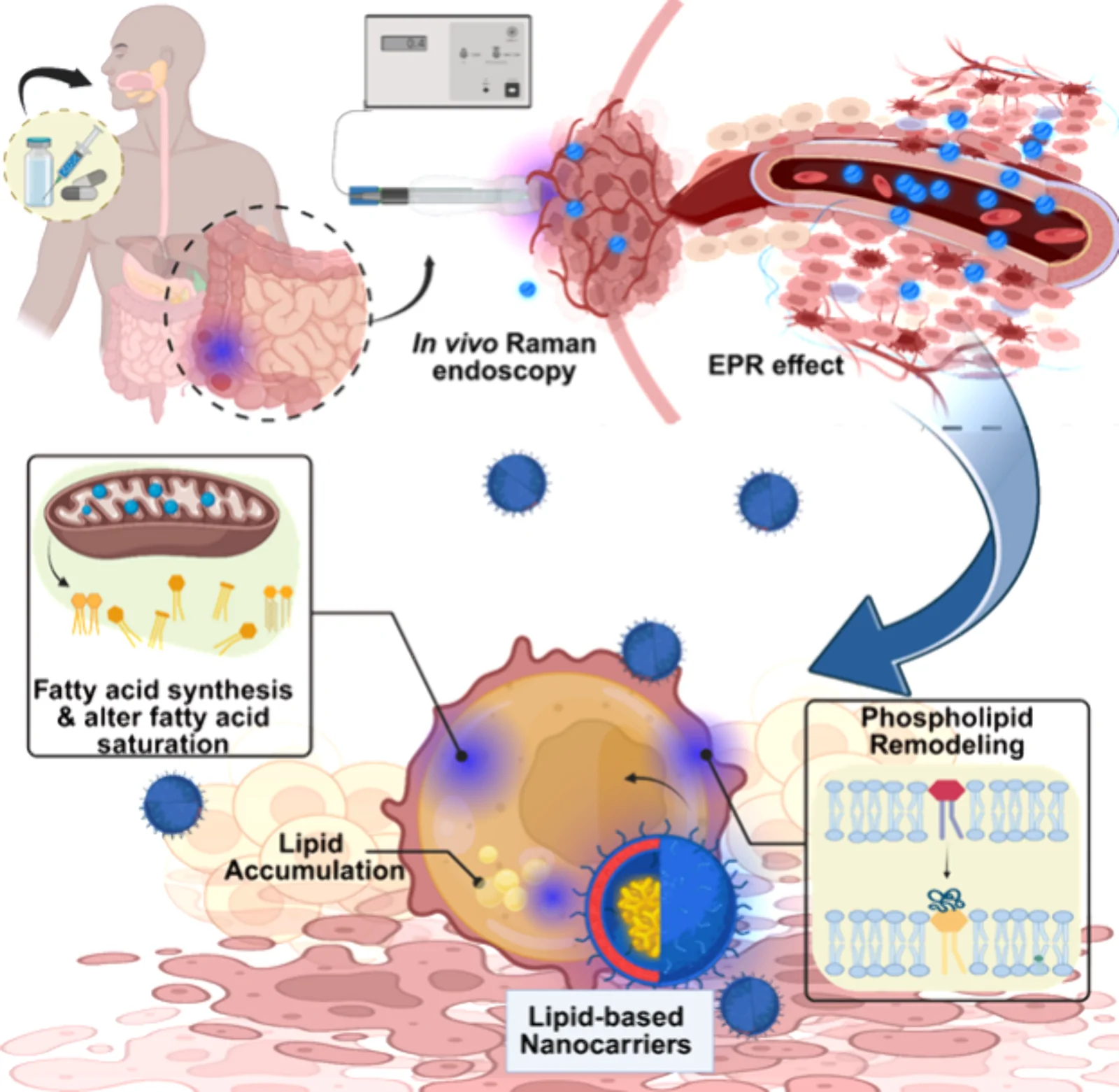
Schematic illustrating Raman nanoprobe-based metabolic imaging of cancer. Engineered nanoprobes incorporating Raman reporters or signal-enhancing elements accumulate preferentially in tumors through the enhanced permeability and retention (EPR) effect, enabling real-time *in vivo* Raman endoscopic detection. Cancer-associated metabolic reprogramming, including increased *de novo* fatty acid synthesis, altered fatty acid saturation, phospholipid remodeling, and lipid droplet accumulation, further enhances Raman signatures that distinguish tumor from normal tissue. Liposomes have enabled drug delivery but are limited by instability and retention, prompting the development of solid lipid nanoparticles (SLNs) with improved stability and retention profiles,^39,56^ and nanostructured lipid carriers (NLCs) with improved stability and retention profiles combine solid and liquid lipids to enhance encapsulation efficiency and physicochemical stability.^74^ Beyond drug delivery, lipid nanocarriers have emerged as versatile optical reporters for intracellular chemical imaging, with tunable membranes that accommodate Raman reporters while minimizing toxic accumulation.^75,76^ Image is created using Biorender.com.

While various polymeric and inorganic matrices offer robust scaffolding, lipid-based nanostructures have emerged as a uniquely powerful class of carriers for metabolic research. Because they are constructed from biomimetic lipid scaffolds,^34,36^ platforms like liposomes,^65^ solid lipid nanoparticles (SLNs),^21,66^ and nanostructured lipid carriers (NLCs)^56^ offer exceptional biocompatibility and versatile loading capabilities for both hydrophobic and hydrophilic cargos. Crucially, by embedding bioorthogonal Raman reporters directly into these lipid matrices, the nanocarrier ceases to be a passive delivery vehicle^67–71^ and instead becomes an active, trackable surrogate for native lipid trafficking.^72,73^ However, background tissue autofluorescence and light scattering can still obscure these weak optical signals if they are not precisely managed.

This review explores how lipid nanoparticle-based Raman probes advance metabolic imaging by incorporating bioorthogonal chemistry, plasmonic enhancement strategies, and multimodal sensing. It highlights innovations such as isotope-labeled Raman reporters and AI-integrated spectral unmixing for data analysis. Further, we recommend standardization of reporting and practice to improve experimental reproducibility and translational capability. Finally, we highlight emerging applications and future directions for Raman probes to perform comprehensive metabolic imaging. To systematically compare the capabilities of various modalities for metabolic imaging, we compiled key performance metrics in Tables 1–3. The comparison of major biomedical imaging modalities for metabolic imaging based on their spatial resolution and molecular sensitivity is presented in Figure 3.

**Table 1:** Comparing Different Metabolic Imaging Modalities Quantitatively

Imaging category	Contrast mechanism	Specificity	Sensitivity	Speed	Spatial resolution
MALDI-MS Imaging	Mass (*m*)-to-charge (*z*) ratio-based detection of ionized metabolites^77^	Very high	Analyte dependent^78^ ^,^ ^b^	∼24 h for brain sections^79,78^ ^,^ ^a^	1.4 μm to 600 nm (for advanced MALDI systems^81,82^ ^,^ ^c^
				∼30 pixel/s^80^ ^,^ ^b^	
LC-MS	Chromatographic separation followed by *m*/*z* ratio detection	Very high	fM^b^	N/A (10–30 min/sample)^83^ ^,^ b	Noninherently^84^
TPF	Two-photon absorption	Moderate (fluorophore dependent)^85^	pM-nM (live/fixed cells)^85^ ^,^ ^b^	Seconds-to-minutes per acquisition^86^ ^,^ ^b^	For TPF-lateral ∼200–500 nm, axial ∼500–1000 nm (diffraction limited)^85,87^ ^,^ ^c^
FLIM	Fluorescence lifetime decay	High for endogenous fluorophores (e.g., NADPH, FAD, collagen, elastin)	Low nM-pM (intracellular fluorophores, probe dependent)^83,88^ ^,^ ^b^	Seconds-to-minutes per image (time-domain FLIM slow, wide-field FLIM at frame rates)^89,90^ ^,^ ^b^	For FLIM: lateral ∼200- 350 nm, axial ∼500–1000 nm^90^ ^,^ ^c^
FRET	Distance-dependent nonradiative energy transfer between donor and acceptor fluorophores^91^	Very high for specific metabolites engineered into the sensors (e.g., glucose, 2-OG, ATP, glutamate)	Dynamic range: low-100 nM to low 10 mM sensor dependent^92^ ^,^ ^b^	Real-time, often milliseconds to seconds depending on readout format^89^ ^,^ ^b^	∼200–400 nm lateral, ∼500–1000 nm axial (diffraction limited)^93^ ^,^ ^c^
Fluorescence microscopy	Targeted fluorescence emission	High for the targeted metabolite or pathway; limited to probe-defined chemistry^93,94^	Highly probe dependent; often pM-nM^95,96^ ^,^ ^b^	Seconds-to-minutes per image in live cells or tissues^97,95^ ^,^ ^b^	∼200–500 nm lateral, 1000 nm axial when imaged via confocal or TPF microscopy^87^ ^,^ ^c^
PET	Detection of gamma photons emitted by positron-emitting radiotracers (positron annihilation γ-rays)	Moderate to high for tracer-defined pathways (glycolysis via FDG, etc.) but indirect; reports “tracer “uptake/flux” rather than direct molecular identity^98^	Very high *in vivo* (pM-nM tracer concentrations in humans; especially single-molecule-equivalent sensitivity at the tracer level per voxel)^98^ ^,^ ^b^	60 min (post FDG injection)^99^ ^,^ ^a^; Imaging: sec–min^100^ ^,^ ^b^	1–5 mm (clinical PET)^101,102^ ^,^ ^b^
MRI	Nuclear magnetic resonance signal contrast from proton density^103^	Moderate (chemical shift)^104^	mM^104,103,105^ ^,^ ^b^	min–hour^106,107^ ^,^ ^b^	0.5 mm^108^ ^,^ ^b^
Photoacoustic microscopy	Optoacoustic pressure waves	Low–moderate^109^	nM−μM^110^ ^,^ ^b^	sec–min,^109^ ^b^	∼200 nm–100 μm^38^ ^,^ ^b^
SHG	χ ^(2)^ Nonlinear optical frequency doubling in noncentrosymmetric materials	ref 111	nM−μM^112^ ^,^ ^b^	Video rate^112^ ^,^ ^c^	<1 μm^113^ ^,^ ^c^
Spontaneous Raman	Inelastic scattering of photons by molecular bonds	High (fingerprint)^113^	∼1 mM (weak signal: 10^7^–10^8^ photon yield)^114^ ^,^ ^b^	Seconds per pixels^115^ ^,^ ^a^	∼500 nm^115^ ^,^ ^c^
SRS	Stimulated loss/gain^116^	High^116^	Sensing limits around mM-sub-Mm (native C–H); higher with engineered tags^117,118^ ^,^ ^b^	μs per pixel (kHz line scan)^115^ ^,^ ^c^	∼300 nm^115^ ^,^ ^c^
CARS	Nonlinear four-wave mixing^119^	High^119^	∼1 mM^120^ ^,^ ^b^	μs per pixel (video rate)^115^ ^,^ ^c^	∼300 nm^115^ ^,^ ^c^
SERS	Plasmon EM/chemical^121^	Very high^122^	nM–pM (10^6^–10^10^ enhancement factors)^123^ ^,^ ^b^	Second per pixel^123,124^ ^,^ ^a^	∼7 nm^125^ ^,^ ^c^
TERS	Plasmon near-field	Very high^126^	fM (10^3^–10^6^ enhancement factors)^127^ ^,^ ^c^	Slow scanning^123^ ^,^ ^a^	10–30 nm^128^ ^,^ ^c^
SORS	Inelastic scattering, offset depth selection^129^	High (fingerprint regions)^130^	∼μM (*in vivo*, targeted)^131,130^ ^,^ ^b^	Seconds per spatial offset^131^ ^,^ ^a^	5 mm^132^ ^,^ ^b^

a Measured value: Empirically derived data from specific experimental setups reported in the literature.

b Typical operating ranges: standard operational parameters generally achieved under standard laboratory or clinical protocols.

c Design targets: ultimate, theoretical, or instrument-limited parameters achievable under fully optimized conditions or specialized systems.

**2:** Comparison of Performance Metrics for Different Modalities

Imaging category	Signal to noise ratio (SNR)	Multiplexing capability	Sample requirement	Penetration depth	Throughput
MALDI-MS Imaging	High^133^	Very high (often hundreds of molecular features in one run)	pg-ng per mm^2^ tissue sections^133^ ^,^ ^a^	Surface-limited (10–20 μm sections)^134,133^ ^,^ ^b^	Low–medium^135^
LC-MS	High (S/N ≥ 3 used for LOD)^136^	Very high (often 100s to 1000s of molecular features^137^	0.2 mg–50 mg wet tissue extract^499,500^ ^,^ ^a^	No spatial penetration (0 μm)^138^	Moderate–high (∼10–30 min/sample)^137^ ^,^ ^b^
TPF	High^139^	High (multifluorophore)^140^	pg–ng (method dependent)^a^	Deeper than confocal (>500 μm to ∼1 mm in living tissue^113^ ^,^ ^c^	Medium^141^
FLIM	Depends on number of photons^141^	Moderate (spectral multiplexing with fluorophores and lifetime-based separation of overlapping species)	Cellular/subcellular levels), fixed cells^142^	With confocal penetration is <100–200 μm in tissue and with TPE -FLIM ∼200–500 μm, deeper with NIR^143^ ^,^ ^b^	n/a
FRET	Moderate to low^144^	High (spectral FRET)^145^	Very small (works at single-cell or subcellular level) (transfected cells)^a^	50–500 μm^144^ ^,^ ^b^	High (live imaging)
Fluorescence microscopy	High^146^	High (multicolor)^147^	very low mass per pixel (nM probe solution)^b^	50–500 μm^148^ ^,^ ^b^	Medium to high (wide field)^b^
PET	Low to moderate^149^	Low in practice because one tracer at a time per PET study^150^	Whole-body possible^151^	Whole body^150^ ^,^ ^b^	Low-to-moderate for patient but high for cohort-based clinical studies
MRI	Moderate^152^	Moderate	None	Whole body^b^	Low
Photoacoustic microscopy	Moderate to high^153,154^	High^155^	None	∼1 mm-3 cm,^38,156^ ^,^ ^b^	High
SHG	Moderate to high^157^	Low^158^	None	<200 μm^113^ ^,^ ^b^	High
Spontaneous Raman	Low^159^	High (broadband)^160^	Solid/liquid^161^	<100 μm^162^ ^,^ ^b^	Low
SRS	Moderate to high^163^	High^161^	Solid/liquid^161^	∼100–300 μm^164^ ^,^ ^b^	High (kHz line scans)^c^
CARS	Moderate to high^165^	Medium	Solid/liquid	<100 μm^162^ ^,^ ^b^	High
SERS	High^166^	Low (hotspots)^167^	Adsorbed on metal	Surface <10 μm for conventional SERS, Upto ∼5 mm in tissue for in vivo SERS probes^168^ ^,^ ^a^	Medium
TERS	Moderate to high^169^	Low	Proximal to tip (∼50 nm gap),^170^ ^,^ ^a^	Surface only (∼10 nm)^171^ ^,^ ^b^	Low
SORS	Low to moderate^130^	High (broadband)^171^	Turbid/subsurface layers	Upto 20 mm (tissue depth)^130^ ^,^ ^c^	Medium (multi-offset scans)

a Measured value: Empirically derived data from specific experimental setups reported in the literature.

b Typical operating ranges: standard operational parameters generally achieved under standard laboratory or clinical protocols.

c Design targets: ultimate, theoretical, or instrument-limited parameters achievable under fully optimized conditions or specialized systems.

**Table 3:** Comparison of Strengths and Limitations of Different Modalities

Imaging category	Label free	Cost of analysis	Strength	Limitation	References
MALDI-MS Imaging	No	Very high ($55/hour)^78^ ^,^ ^a^	Direct spatial metabolomics profiling	Matrix effects, no in-depth information	^78^
LC-MS	Yes	Very high (∼$50–$300 per sample)^24^ ^,^ ^a^	Gold standard for metabolites quantification, excellent dynamic range, broad coverage of metabolites	No spatial information	^24^
TPF	Probe dependent	∼$100s to $1000s (pulsed ps or fs lasers used)^113^ ^,^ ^b^	Higher spatial resolution and penetration depth than mass spectrometry	Photodamage, fluorophores needed	^113^
FLIM	Yes	High as it requires a confocal or TPF, pulsed source and time-resolved detection^172^ ^,^ ^b^	High spatial resolution, largely concentration independent	Limited to fluorophores only, photon-starved areas lead to noisy lifetime maps, quantification more demanding than MALDI	^172^
FRET	Probe dependent	Medium^144,172^ ^,^ ^b^	Real-time dynamic	Multiplexing is limited	^144^,^172^
Fluorescence microscopy	Probe dependent	Low–medium^173^ ^,^ ^b^	Targeted probes	Photobleaching	^173^
PET	Probe dependent	Very high ($M)^174^ ^,^ ^b^	Whole-body functional imaging	Radiation source, spatial resolution is limited	^174^
MRI	Label free	High ($M)^108,174^ ^,^ ^b^	3D metabolic maps	Low sensitivity	^108,174^
Photoacoustic microscopy	Label free	$10K–100K^100^ ^,^ ^b^	Higher penetration depth than fluorescence imaging	Limited functional imaging capability	^100^
SHG	Label free	High^113^ ^,^ ^b^	Label-free imaging of collagen 1	Limited functional imaging capability	^113^
Spontaneous Raman	Label free	Medium^115^ ^,^ ^b^	Chemical imaging with wide coverage of molecules	Weak signal, slow	^115^
SRS	Label free	High^115^ ^,^ ^b^	Quantitative and video rate imaging	Limited multiplexing capability, challenging to acquire a broad full spectrum in a single acquisition	^115^
CARS	Label free	High^115,113^ ^,^ ^b^	Fast video rate imaging	Nonresonant background distorts the Raman peak position (does not match the peak position of spontaneous Raman)	^115,113^
SERS	Probe dependent	High^176,124^ ^,^ ^b^	Ultrasensitive	Reproducibility is limited; low dynamic range for concentrations	^176,124^
TERS	Probe (tip dependent)^177^	High^178^ ^,^ ^b^	Nanoscale spatial resolution ^c^	Slow, very small spatial range	^177,178^
SORS	Label free	High (offset optics needed)^179^ ^,^ ^b^	Noninvasive; chemical analysis at high penetration depth	Demands depth-calibration modeling	^179^

a Measured value: Empirically derived data from specific experimental setups reported in the literature.

b Typical operating ranges: standard operational parameters generally achieved under standard laboratory or clinical protocols.

**Figure 3:**
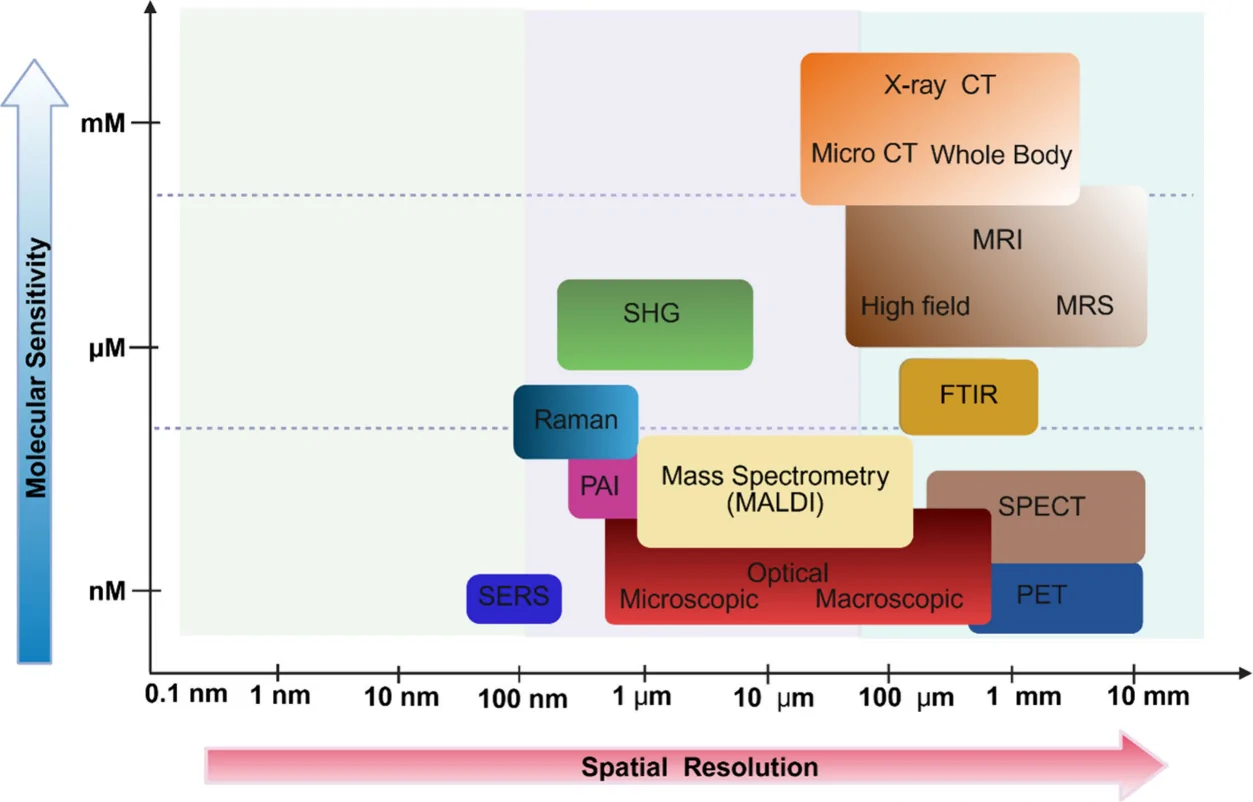
Comparison of major biomedical imaging modalities for metabolic imaging based on their spatial resolution and molecular sensitivity.

## Comparison of Different Tools Available for Metabolic Imaging

### Different Raman Spectroscopy Methods

Raman spectroscopy (first described in 1928 ^180^) originates from the inelastic scattering of light interacting with the vibrational modes of molecular bonds. When a monochromatic laser in the visible, near-infrared, or near-ultraviolet range interacts with the sample^162^, the incident photons interact with molecular bonds and can induce transitions between vibrational energy states. When a molecule absorbs part of the energy from the incoming pump photon (ω_p_), it is promoted to a higher vibrational level. As a result, the scattered photons lose energy and appear at a lower frequency than the excitation light; such photons are referred to as Stokes photons (ω_s_). Conversely, if the incident photon interacts with a molecule that is already in an excited vibrational state, energy may be transferred from the molecule to the photon. In this case, the scattered photon gains energy and is emitted at a higher frequency than the excitation light, producing anti-Stokes (ω_as_). The energy difference between the incident and scattered photons is known as the Raman shift, which corresponds directly to the vibrational frequency of the molecular bond. Raman shift is expressed as follows: $\Delta \omega \;\left({cm}^{-1}\right)=\left[\frac{1}{\lambda_{p}}-\frac{1}{\lambda_{s}}\right]\times 10^{7}$ . Here, λ_*p*_ and λ_*s*_ are in nm, and 10^7^ is the conversion factor for centimeters to nanometers.

In spontaneous Raman spectroscopy, a single laser beam at the pump frequency (ω_p_) illuminates the sample, generating scattered photons at Stokes (ω_s_) and anti-Stokes (ω_as_) frequencies through inelastic scattering. Although this approach provides chemically specific information, spontaneous Raman scattering is intrinsically inefficient, with a photon conversion efficiency typically 1 in every 10^8^.^181^ As a result, signal intensities are extremely weak and often require long acquisition times, limiting the applicability of spontaneous Raman spectroscopy for rapid or live biological imaging.

To address these limitations, Coherent Raman scattering (CRS) spectroscopy was developed to enhance signal generation through nonlinear optical processes. CRS techniques employ two synchronized pulsed laser beams, typically in the picosecond or femtosecond regime, with frequencies corresponding to the pump (ω_p_) and Stokes (ω_s_) beams. Typically, the pump laser is tunable from 700 to 990 nm via optical parametric oscillator, while the Stokes beam uses a fixed wavelength such as 1031, 1040, or 1064 nm. The two beams are spatially and temporally overlapped and focused on the sample. When the frequency difference between these beams matches the vibrational frequency of a molecular bond (Ω_vib_), the molecular vibration is coherently driven. This results in a substantial amplification of the Raman signal. Among the CRS approaches, coherent anti-Stokes Raman Scattering (CARS) and stimulated Raman scattering (SRS) spectroscopy represent the most widely used imaging modalities. Even though, coherent Stokes Raman scattering (CSRS) has been demonstrated for microscopic uses.^182^ The pump beam wavelength can then be determined from the following equation : $\lambda_{p}\;\left(nm\right)=\frac{1}{\Delta \omega \;\left({cm}^{-1}\right)\times \frac{cm}{10^{7}\mathrm{nm}}+\frac{1}{\lambda_{s}\left(\mathrm{nm}\right)}}$

In coherent anti- Stokes Raman scattering (CARs, first described in 1965 ^183^) spectroscopy, two pulsed laser beams tuned to the pump and Stokes frequencies interact with the sample. CARS generates an anti-Stokes scattering signal via four-wave mixing between the incident pump and the lasers. When the difference between these frequencies matches the vibrational frequency Ω_vib_, a nonlinear optical interaction coherently excites the molecular vibration. The excited vibration subsequently interacts with another pump photon, producing a coherent anti-Stokes photon (ω_as_). This anti-Stocks signal occurs at a higher frequency (shorter wavelength) than the excitation beams, allowing it to be readily separated from the excitation light and detected using a photomultiplier tube (PMT) after passing through appropriate optical filters. To selectively image specific chemical species, the pump wavelength can be tuned using an optical parametric oscillator (OPO). This ensures that the frequency difference between the pump and Stokes beams corresponds to the Raman shift of the target vibrational mode. Despite its advantages, CARS imaging can be affected by a nonresonant background signal arising from nonlinear electronic responses within the focal volume. Interference between this background signal and the resonant vibrational signal may distort spectral features and limit the sensitivity and specificity of detection.

Stimulated Raman scattering (SRS, first described in 1962 ^184^) spectroscopy provides an alternative CRS approach that enables highly sensitive vibrational imaging with improved spectral fidelity. In SRS, when the frequency difference between the ω_p_ and ω_s_ beams matches a Ω_vib_, nonlinear interaction between the beams stimulates molecular vibrations and facilitates energy transfer between them. During this process, a pump photon is effectively converted into a Stokes photon. Consequently, the pump beam experiences a decrease in intensity, known as stimulated Raman loss (SRL), while the Stokes beam undergoes a corresponding increase in intensity, referred to as stimulated Raman gain (SRG). These small intensity changes provide the vibrational contrast used for SRS imaging. Although SRS provides chemically specific contrast, the associated SRG and SRL signals are extremely small, typically in the order of ΔI/I < 10^–7^ to 10^–4^ in the pump or Stokes beam.^185^ Importantly, if the frequency difference between the pump and Stokes beam does not correspond to a molecular vibration, no energy transfer occurs, and neither SRL nor SRG signals are generated. Unlike CARS, SRS does not produce a nonresonant background, allowing more accurate spectral measurements. Even though SRL and SRG signals are specific to molecular bonds, they are faint and often overshadowed by laser noise fluctuations, posing a major hurdle for SRS microscopy implementations. Since most laser noise occurs at relatively low frequencies, generally below 1 MHz.^186^ SRS employs high-frequency modulation techniques to isolate the Raman signal. In a typical configuration, the Stokes beam is modulated at frequencies exceeding 2 MHz, while the pump beam remains unmodulated. When the beams interact with the sample under resonance conditions, the resulting SRL or SRG signal appears at the same modulation frequency. This modulated signal can then be selectively extracted and amplified using a lock-in amplifier, enabling highly sensitive detection of Raman signals.^184^ An additional advantage of SRS is its capability for quantitative chemical imaging. The intensity of the SRS signal (S) depends on several factors, including the pump beam intensity (I_P_), Stokes beam intensity (I_S_), analyte concentration ([c]), and the molecular Raman cross-section (σ). For a given analyte, the signal follows the proportional relationship:

$$S\propto \left[c\right]\times \sigma \times I_{P}\times I_{S}$$

This linear dependence allows direct quantification of molecular concentrations, making SRS a powerful platform for investigating chemical composition and metabolic processes in complex biological systems.^187^ SRS spectra align closely with conventional Raman spectra, allowing for straightforward sample analysis. SRS achieves detection limits in millimolar concentrations for native biomolecules, such as C–H vibrations from proteins and lipids. Bioorthogonal Raman tags have boosted sensitivity using Raman-active groups like alkyne and nitriles, while boasting high scattering cross sections; plasmonic nanostructures and electronic preresonance from organic dyes have also amplified SRS signals.^115^

Surface-Enhanced Raman scattering (SERS, first observed in 1974 ^188–190^ ) and tip-enhanced Raman scattering (TERS, first observed in 2000 ^191–194^ ) amplify Raman signals using plasmon effects. SERS uses rough metal surfaces or NPs to enhance signals via electromagnetic and chemical mechanisms. TERS employs a plasmonic nanotip 50 nm above the sample’s region of interest to confine light at the nanoscale, achieving 10–30 nm spatial resolution and localized enhancement.^128^ Spatially offset Raman spectroscopy (SORS) is a nondestructive optical technique that probes subcellular molecular composition in scattering or layered samples. By collecting Raman signals at a spatial offset from the laser excitation, SORS can detect chemicals beneath the surface of tissues, powders, or opaque containers without sample preparation. It provides molecular fingerprints for the identification and monitoring of dynamic or buried materials. Figure 4 shows different Raman-based imaging modalities and representative experimental configurations.

**Figure 4:**
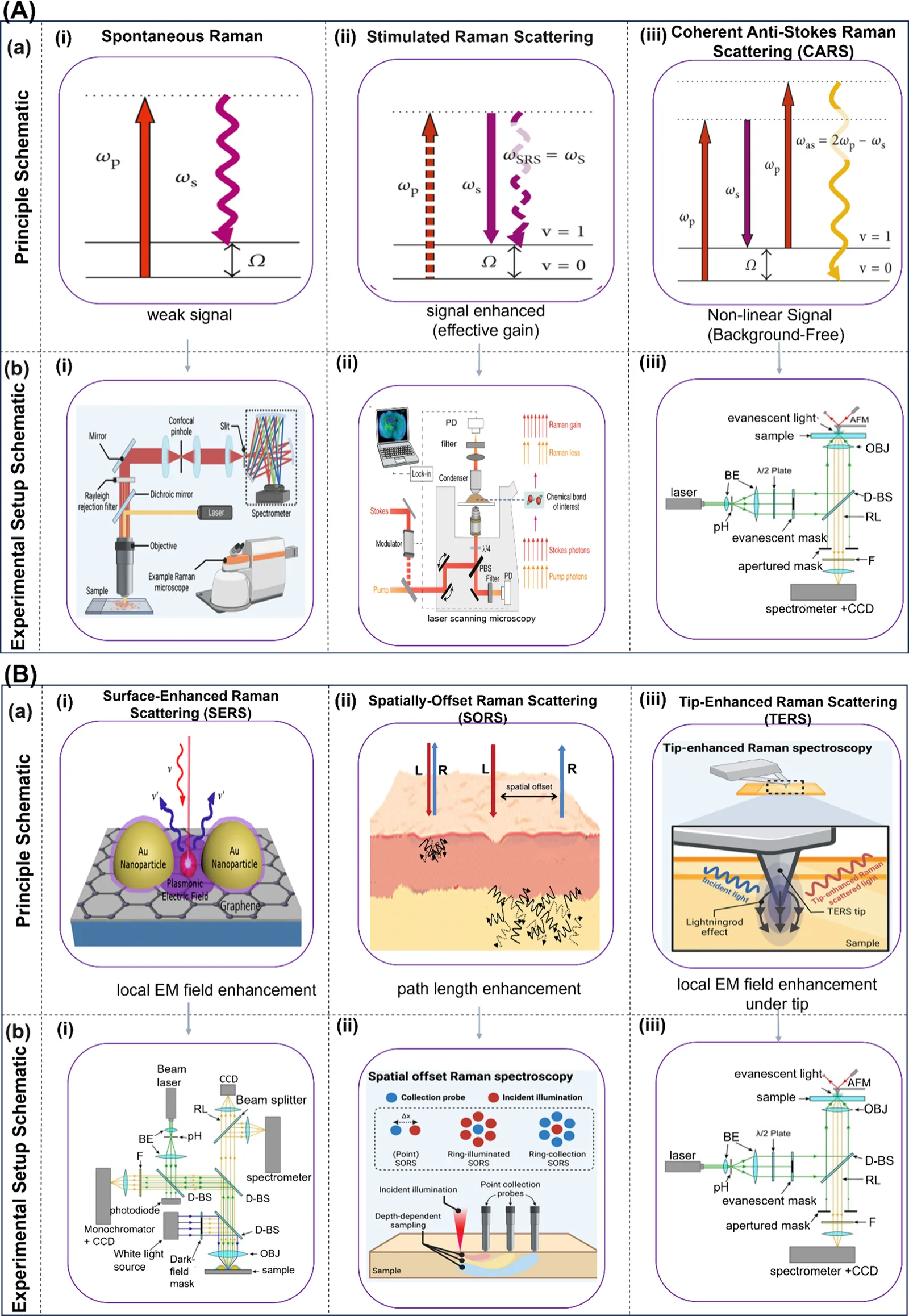
Comparison of Raman-based imaging modalities and representative experimental configurations. (A) Schematic principles (a) and instrument setups (b) for (i) spontaneous Raman scattering, (ii) stimulated Raman scattering (SRS), and (iii) coherent anti-Stokes Raman scattering (CARS). Panel (A)-(b-i) and Panel (B)-(a-iii) and (b-ii) adapted with permission from ref 178 (Copyright 2024 Wiley-VCH GmbH). Available under CC-BY 4.0 License. Panel (A)-(b-ii) adapted with permission from ref 195 (Copyright 2019 Springer Nature Limited). Panel (A)-(a-i, ii, and (iii) reproduced from ref 176. Available under CC-BY 4.0 License. Panel (B) Schematic principles (a) and representative configurations (b) for (i) surface-enhanced Raman scattering (SERS), (ii) spatially offset Raman spectroscopy (SORS), and (iii) tip-enhanced Raman scattering (TERS). Panel (B)-(a-i) adapted with permission from ref 196 (Copyright 2017 Elsevier Ltd.). Panel (B)-(a-ii) adapted with permission from ref 197 (Copyright 2013 Royal Society of Chemistry) and ref 198 (Copyright 2017 American Chemical Society). Panel (A)-(b-iii) and Panel (B)-(b-i and iii) adapted with permission from ref 128 (Copyright 2019 Fundamentals and Frontiers. *Nanoscale Res*. *Lett*). Available under CC-BY 4.0 License.

## Multimodal Imaging Systems

Raman spectroscopy provides exceptional molecular specificity, but its sensitivity is limited by optical attenuation and scattering. Coupling Raman spectroscopy with other modalities, such as photoacoustic (PA) imaging, overcomes this constraint by combining chemical precision with deep-tissue sensitivity. PAI imaging is based on the photoacoustic effect, where absorbed light energy is converted into heat. When a specimen is illuminated with a short laser pulse, typically lasting a few nanoseconds, light-absorbing molecules absorb the incident optical energy. This absorption promotes electrons from their ground state to higher-energy excited states. The excited electrons then rapidly relax back to the ground state, releasing the absorbed energy through different pathways.^199^ The nonradiant dissipation of this energy generates a transient temperature increase, resulting in thermoelastic expansion and the production of acoustic pressure waves. The magnitude of the initial pressure rise is proportional to the local optical absorption coefficient, laser fluence, and Grüneisen parameter, thereby providing optical absorption contrast from deep tissue regions.^200^

Another approach is to combine Raman imaging with fluorescence lifetime imaging (FLIM) and two-photon microscopy (TPM) (Figure 5). Rather than monitoring fluorescence intensity alone, which is susceptible to photobleaching and concentration artifacts, FLIM measures the nanosecond decay dynamics (excited-state lifetime) of NADH and FAD. Because these lifetimes shift based on whether the coenzymes are free or protein-bound, FLIM provides a sensitive, noninvasive readout of cellular metabolic states. It serves as a direct optical counterpart to the nonradiative pathways.^201^ Alternatively, the absorbed energy can be dissipated as heat through nonradiative relaxation processes. In most cases, both mechanisms occur concurrently, with the fraction of energy allocated to fluorescence emission versus thermal dissipation depending on the absorber’s properties. The fraction of absorbed energy converted into heat, referred to as the photothermal conversion efficiency, which can be described by

1 $$\sigma =1-\phi =\frac{k_{h}}{k_{f}+k_{h}}$$

where σ represents the efficiency of heat generation, φ denotes the fluorescence quantum yield, *k*_*f*_ corresponds to the fluorescence decay rate constant, and *k*_*h*_ represents the rate constant for nonradiative heat dissipation. A higher photothermal conversion efficiency results in stronger photoacoustic signal generation. The resulting acoustic waves propagate through biological tissue and are detected by ultrasound transducers. The acquired signals are subsequently reconstructed into images using computational algorithms that operate on principles like those employed in conventional ultrasound imaging systems.^202–207^ A major advantage of PAI over fluorescence-based techniques arises from its hybrid optical-acoustic detection strategy. PAI converts optical absorption into ultrasonic waves, which experience significantly less scattering than photons during tissue propagation. Consequently, PAI can visualize structures several centimeters beneath the tissue surface while maintaining spatial resolutions on the order of hundreds of micrometres. The strength of the generated photoacoustic signal is governed by several physical parameters and can be described as^208^

2 $$P\propto \Gamma \left(T\right)\cdot \sigma {.\mu }_{a}\cdot F$$

where P is the initial pressure of the PA waves, Γ­(*T*) is the Grüneisen parameter at the local temperature (T), σ is the heat conversion efficiency described in eq 1, μ_*a*_ is the optical absorption coefficient, and F is the optical fluence. Enhancement of any of these parameters can increase the amplitude of the detected photoacoustic signal. In practical applications, signal enhancement is primarily achieved by optimizing optical absorption and light delivery. However, increasing laser fluence is constrained by maximum permissible exposure (MPE) safety limits and often leads to nonselective signal amplification throughout the tissue, providing only limited improvement in image contrast,^209^ Consequently, considerable efforts have focused on developing exogenous contrast agents with strong optical absorption characteristics to selectively enhance photoacoustic signals. Such contrast agents have been widely employed in biomedical applications, including cancer detection, lymphatic imaging, and real-time monitoring of drug delivery processes. Because photothermal conversion efficiency is inversely related to fluorescence quantum yield, absorbers exhibiting lower fluorescence emission generally produce stronger photoacoustic responses, making them particularly attractive for PAI applications.^199^

**Figure 5:**
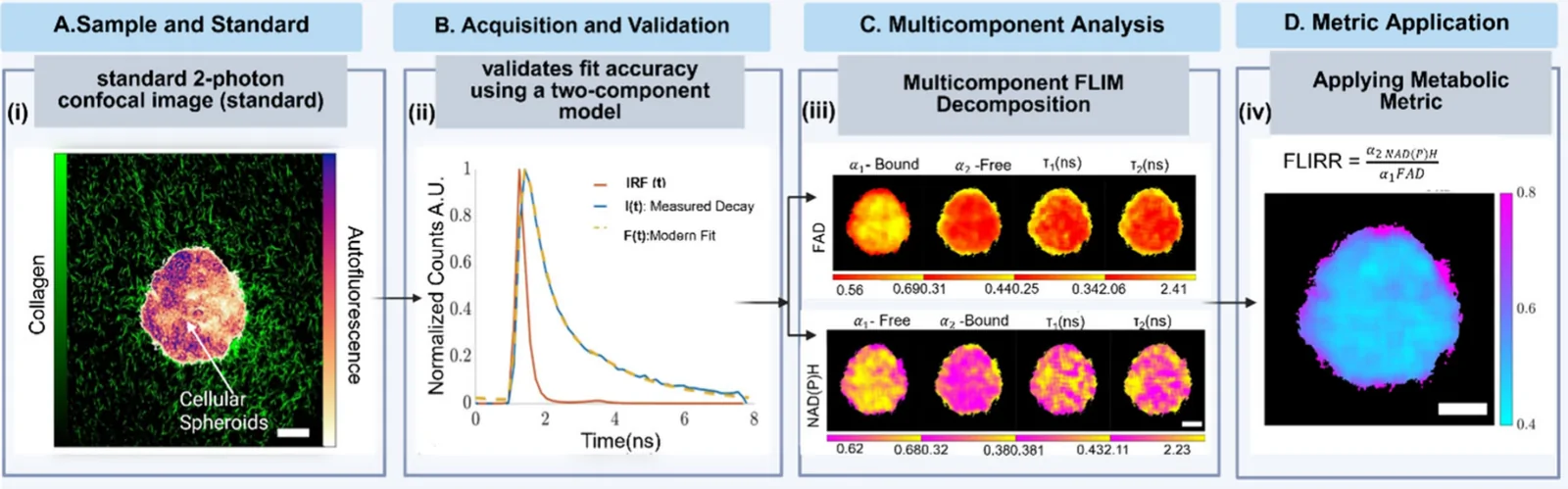
Fluorescence lifetime imaging microscopy (FLIM) methodology and analysis. (A) (i) Overlay of time-integrated NAD­(P)H autofluorescence and second harmonic generation (SHG) collagen signals in an MCF-10A spheroid. (B) (ii) Fluorescence decay curve, I­(t), modeled by convolution of the instrument response function (IRF) with a biexponential decay and fitted using nonlinear least-squares analysis. (C) (iii) Extracted lifetime parameters (τ_1_, τ_2_, α_1_, and α_2_) for NAD­(P)H and FAD. (D) (iv) FLIRR map generated from the ratio of bound NAD­(P)H to bound FAD. Scale bar = 100 μm. Reproduced from ref 201 (Copyright 2023 Springer Nature). Available under a CC by 4.0 License.

Lipid-based nanostructures enhance the PA sensitivity and therapeutic precision. For example, near-infrared (NIR)-activated ruthenium phthalocyanine-loaded lipid nanoparticles have achieved deep-tissue targeting and strong PA contrast for guided photothermal therapy, underscoring the versatility of lipid matrices in Raman-PA multimodal systems.^210^ Incorporation of lipid-based Raman-active nanostructures further enhances optical absorption and acoustic conversion efficiency.^211^ Recent studies have validated this multimodal concept. A group of researchers have developed dual-ratiometric SERS-PA nanosystems for quantitatively visualizing hydrogen peroxide dynamics in cancer with subcellular precision.^212,213^ Studies have also designed a Cu^2+^-responsive nanogapped gold nanoprobe functionalized with PNIPAM and dual Raman reporters that allow NIR-II PA and ratiometric SERS detection of copper ions *in vivo*.^214^

Additionally, recent research has also reported the development of MRI-PA-Raman triple-modality nanoparticles that enabled intraoperative brain tumor delineation with high anatomical and chemical accuracy.^215^ Magnetic resonance imaging (MRI) is a noninvasive, nonionizing technique that visualizes physiological and pathological features by probing water proton relaxation. It provides deep-tissue imaging with high soft-tissue contrast and spatial resolution (∼50 μm). Molecular sensitivity is enhanced by T_1_ and T_2_ contrast agents (T_1_-positive) or (T_2_-negative).^216,217^ Efficient T_1_ probes require high coordinated water molecules, optimal water residence times, and slow nanoparticle rotational dynamics. Contrast agents enhance MRI sensitivity by incorporating paramagnetic ions such as Gd­(III) or Mn­(II) into nanostructures to increase water proton relaxation rates.^217^ T_2_ agents such as SPIONS and USPIONS produce negative contrasts via magnetic susceptibility and depend on particle size, crystalline properties, and surface chemistry.^218^ Coupling Raman spectroscopy with MRI integrates Raman’s molecular precision with MRI’s anatomical depth, enabling comprehensive chemical and structural assessment for improved diagnostics and therapy monitoring.^219^

Recently, ultrasmall metal ferrite nanoparticles (MFe_2_O_4_, M = Fe, Zn or Ni) have demonstrated dual T_1_/T_2_ performance, with high r_1_ relaxivity, low r_2_/r_1_ ratios (where r_2_ transverse relaxivity), and prolonged blood circulation. This combination enables high-resolution vascular imaging. Integration with lipid-based or Raman-active platforms allows multimodal chemical and anatomical imaging.^218,220^ Advances in ^19^F MRI have further expanded multimodal imaging through probes combined with fluorescence, photoacoustic imaging, and SERS.^221^ In addition, substrate-free Raman-PET nanoprobes, such as [^18^F]-AS-DPPT-TT NPs, allow ultrasensitive Raman detection with preoperative PET localization for precise tumor boundary mapping.^222^

While these optical and acoustic modalities offer real-time, nondestructive volumetric mapping, they remain limited in resolving complex, overlapping molecular species as discussed in the previous section. To validate these optical signatures with structural precision, MALDI can be integrated into an analytical workflow. The integration of MALDI and Raman provides highly multiplexed *m*/*z* tracking of thousands of individual lipids and metabolites across an *ex vivo* section (Figure 6). Consequently, while Raman and PAI offer deep-tissue, nondestructive visualization of spatial architecture *in vivo*, MALDI-MSI also serves as a complementary tool for identifying the precise molecular species underlying the observed optical contrast **(** Figure 7 **)**.

**Figure 6:**
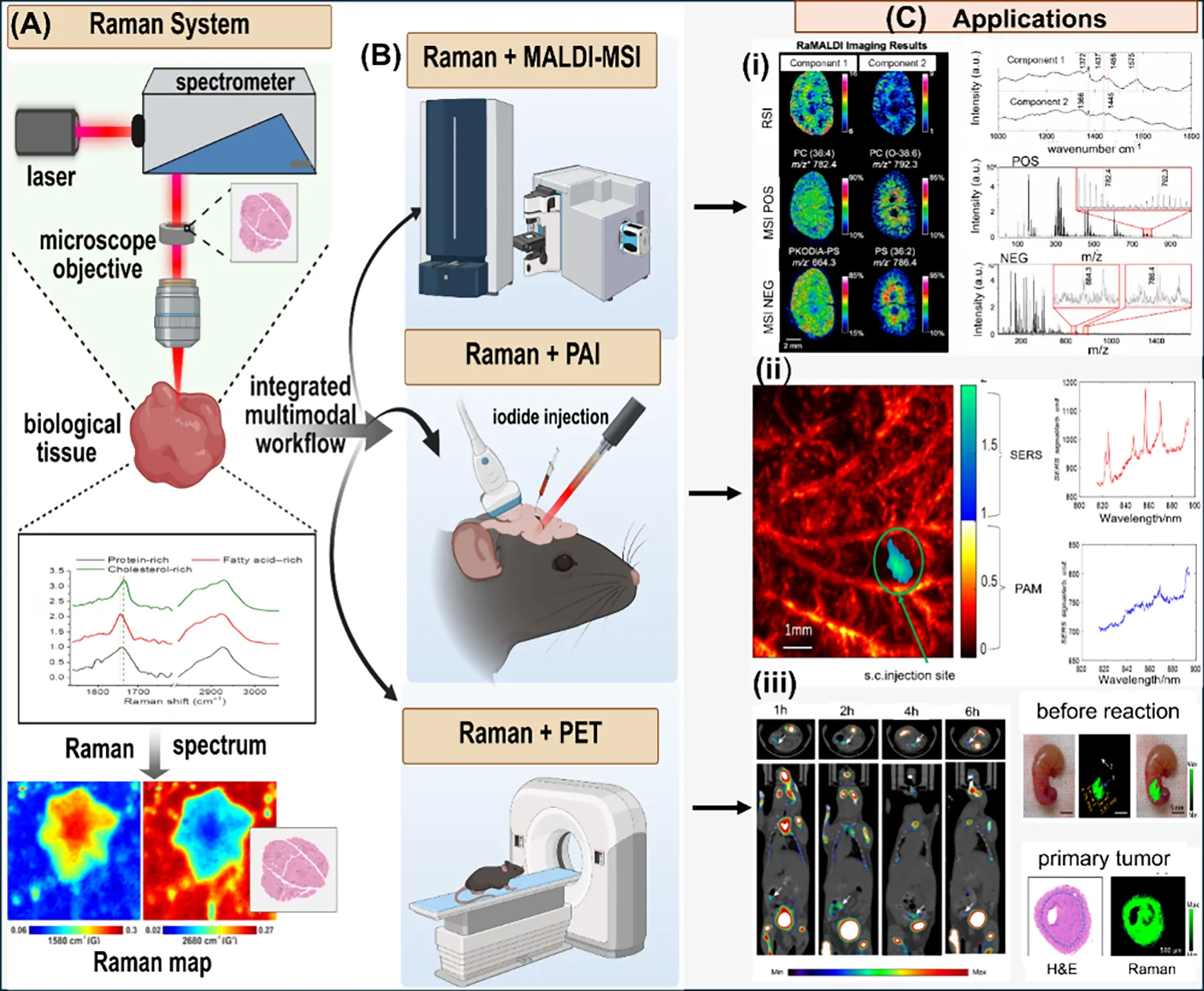
Integrated Raman-based multimodal imaging and applications. (A) Schematic of a Raman microscope and tissue imaging workflow, illustrating acquisition of Raman spectra and chemical maps. (B) Integration of Raman imaging with matrix-assisted laser desorption/ionization mass spectrometry imaging (MALDI-MSI), photoacoustic imaging (PAI), and positron emission tomography (PET) to provide complementary molecular and anatomical information. (C) Representative multimodal applications: (i) Raman–MALDI (RaMALDI) imaging of brain tissue showing MCR-ALS components, K-means clustering, lipid distributions (PC 38:6, PC 40:4, PI 18:0/20:4, and C24:1 sulfatide), and corresponding Raman/MSI spectra (scale bar: 2 mm; adapted with permission from ref 25, Copyright 2023 Elsevier B.V.), (ii) Raman–PAI demonstrating coregistered imaging of rat ear vasculature, targeted HeLa cells, and SERS detection in chicken embryo membranes (adapted with permission from ref 223, Copyright 2016 SPIE) (Available under a CC by 4.0 License), (iii) Raman–PET combining whole-body PET imaging with intraoperative Raman-guided tumor resection and histological validation in a mouse colon cancer model (adapted with permission from ref 222, Copyright 2025 American Chemical Society).

**Figure 7:**
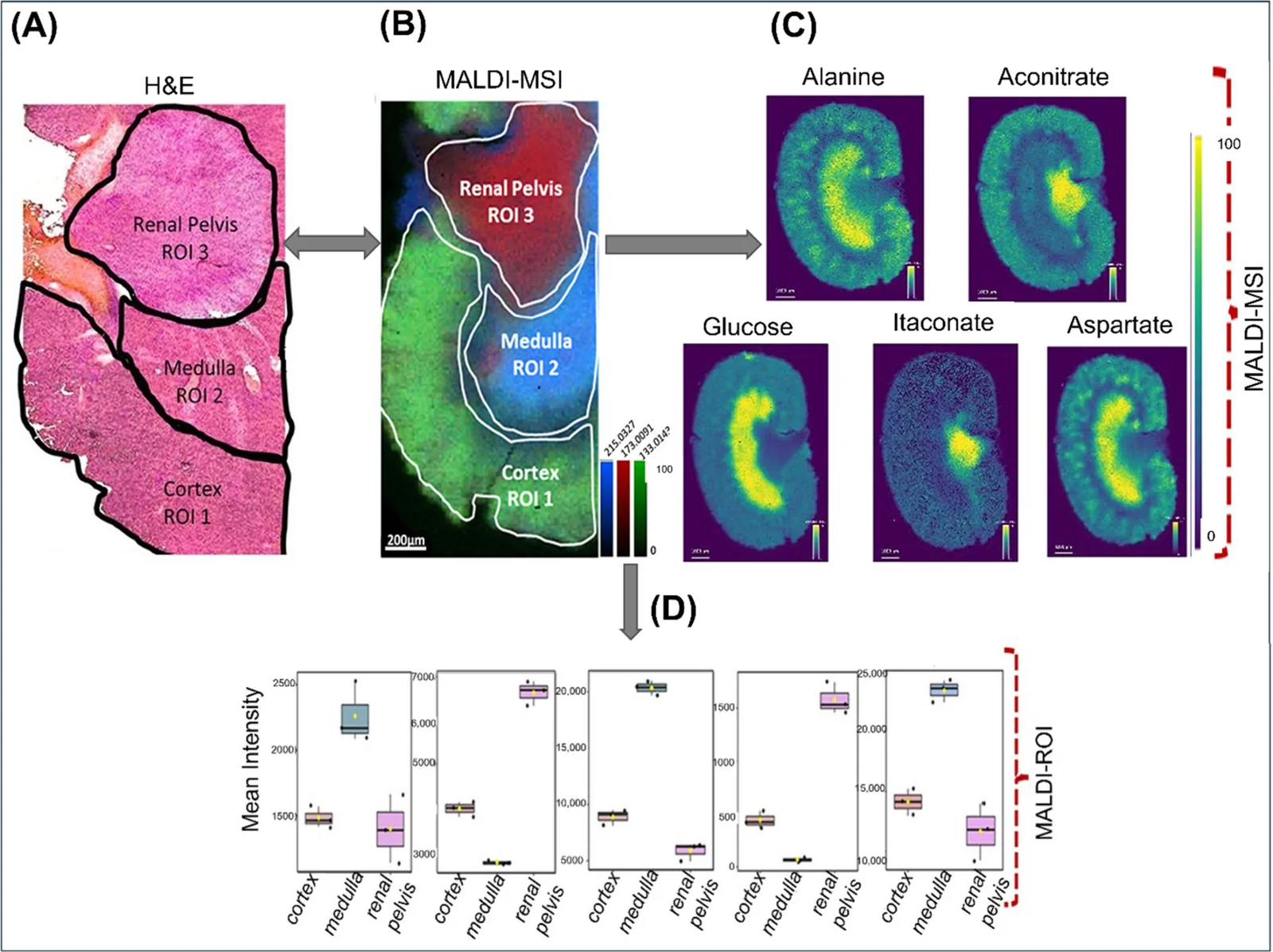
Integrated spatial metabolomics of kidney tissue using MALDI mass spectrometry imaging (MSI). (A) H&E-stained kidney section after basic hexane treatment showing tissue architecture. (B, C) Overlayed MSI ion maps of glucose, malate, and itaconate following basic hexane washing (scale bar: 1 mm). (D) Boxplots of normalized ion intensities across MALDI-defined regions of interest (ROIs), validating the spatial metabolite distributions. Adapted with permission from ref 201 (Kasarla et al., *Anal. Chem.* 2025, 97, 10720–10728. Copyright 2025 American Chemical Society). Available under CC-BY 4.0 License.

### Nanoparticle Engineering for Metabolic Imaging

Growing recognition of cellular heterogeneity has reshaped our understanding of tissue physiology and disease.^224^ Single-cell transcriptomic and proteomic studies show that phenotypically similar cells can exhibit distinct metabolic states.^162^ Fluorescence-based assays typically reveal proteins but provide limited information on small-molecule metabolism.^225,226,164^ Raman spectroscopy can overcome these limitations by imaging lipids and metabolites without labels.^227,228^ Further, lipid-based nanocarriers incorporating vibrational tags can further convert metabolic changes into quantifiable Raman fingerprints.^162,229,230^

## Lipid-Based Nanocarriers

Lipids form the structural foundation of cellular membranes and organellar compartments.^231,232^ Their dynamic roles in signaling,^233^ energy storage, and trafficking make them ideal substrates for monitoring metabolic transitions.^234^ Because Raman scattering depends on polarizability of the molecule, embedding tags in lipids alters signals by local liquid-crystalline order.^235,236^ Consequently, lipid Raman nanostructures function both as biocompatible delivery vehicles and as sensitive reporter systems reflecting their immediate biochemical milieu.

To achieve this multifunctionality, lipid-based Raman tag systems typically integrate three modular pillars: a lipid scaffold, an encapsulated reporter, and a plasmonic core for signal enhancement. For example, in the “LipoGold” platform, gold NP clusters are assembled onto lipid vesicles containing Raman reporters such as 4-Mercaptobenzoic acid, 4-MBA),^70^ while specific Raman reporters are embedded directly into lipid bilayer.^70^ Within this configuration, the lipid shell provides structural stability, biocompatibility, and tight payload encapsulation.^71^ Concurrently, the integrated metallic NPs (such as silver,^237^ gold^71^) serves as a plasmonic core that generates localized electromagnetic “hotspots”. Similarly, lipid-polymer hybrid NPs combine a polymeric core, like PLGA, for the payload loading.^238^ When laser excitation matches the surface plasmon resonance, these hotspots amplify Raman scattering by orders of magnitude, overriding the inherently weak scattering cross-section of the embedded reporters. This helps achieve single-particle detection sensitivity. Beyond plasmonic metals, carbon-based NPs offer distinct advantages, such as optical reporters or stabilizing agents in lipid-based nanocarriers. For example, carbon nanotubes have been used to stabilize nanostructured lipid nanoparticles (LNPs) as optical reporters for endolysosomal lipid content via photoluminescence.^239^ These modular designs thus allow the system to function dually as a delivery vehicle and metabolic reporter molecules.^70^

Liposomes are spherical vesicles composed of one or more phospholipid bilayers surrounding an aqueous core (Figure 8), enabling the encapsulation of both hydrophilic and hydrophobic cargos. In contrast, solid lipid nanoparticles (SLNs) consist of a solid lipid core stabilized by surfactants, providing improved physical stability and protection of encapsulated molecules but often suffer from limited drug-loading capacity due to their highly ordered crystalline structure. Nanostructured lipid carriers (NLCs) were developed as a second-generation lipid nanoparticle system by incorporating liquid lipids into the solid lipid matrix, creating structural imperfections that increase the payload loading, reduce drug expulsion during storage, and improve formulation stability. More recently, lipid nanoparticles (LNPs), composed of ionizable lipids, helper phospholipids, cholesterol, and PEGylated lipids, have emerged as highly efficient carriers for nucleic acid delivery and have gained widespread attention through mRNA vaccine technologies. These differences in architecture influence cargo loading, biodistribution, cellular uptake, and suitability for Raman imaging applications, making carrier selection an important consideration for metabolic imaging and theranostic applications.

**Figure 8:**
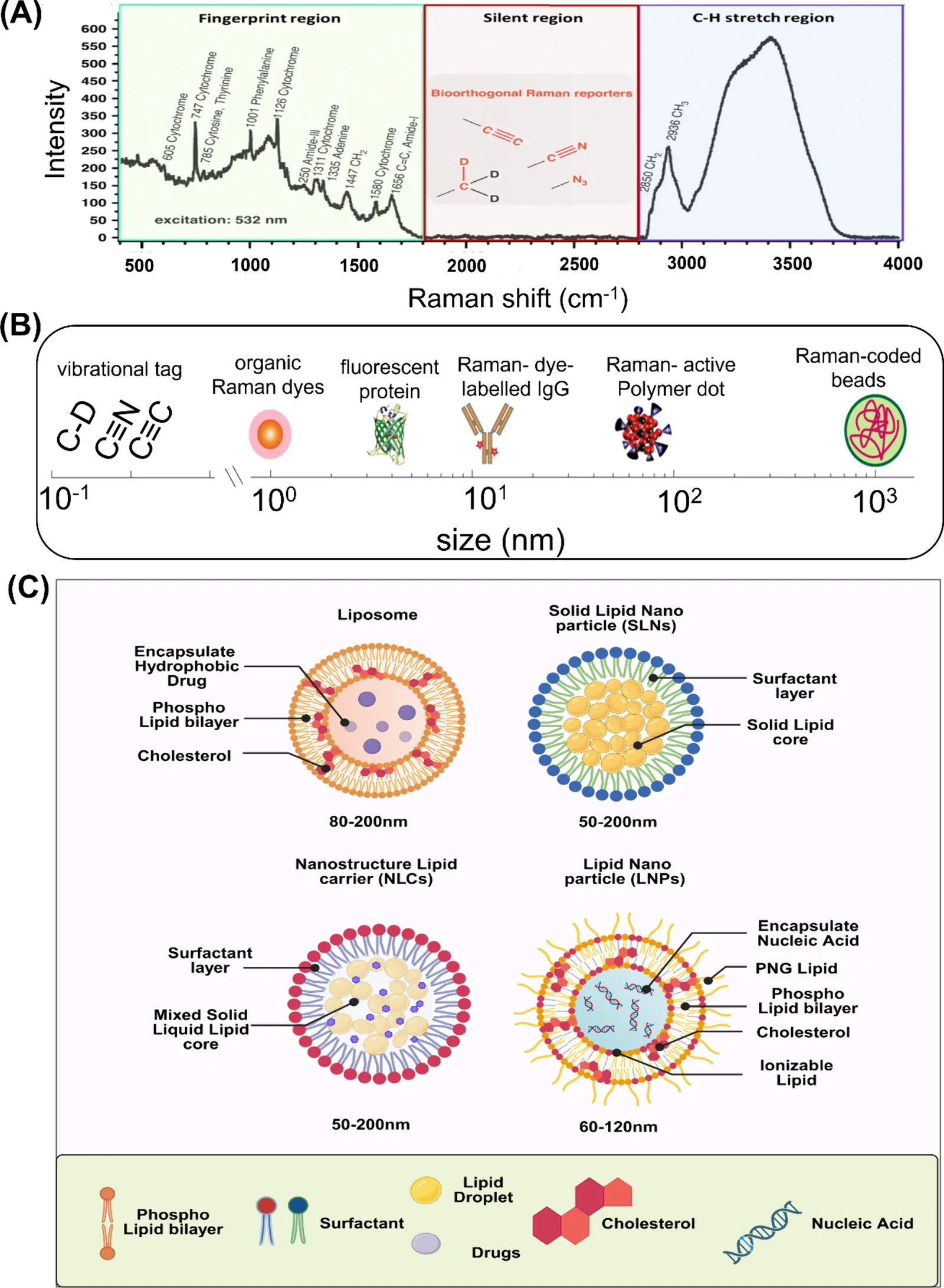
Conceptual framework of Raman-active lipid nanoparticles (NPs) for metabolic imaging. Panel (A) Representative Raman spectrum highlighting the fingerprint (400–1800 cm^–1^), cell-silent (1800–2800 cm^–1^), and C–H stretching (2800–3800 cm^–1^) regions, with bioorthogonal reporters (alkynes (C≡C), nitriles (C≡N), azides (N_3_), and deuterium (C–D) labels) producing distinct signals in the cell-silent window for background-free detection (adapted with permission from ref 243, Copyright 2024 Royal Society of Chemistry, Available under CC-BY 3.0 License). Panel (B) Schematic of stimulated Raman scattering (SRS) probes, including small vibrational tags, Raman dyes, polymer dots, and spectrally encoded beads for multiplex imaging (adapted with permission from ref 195, Copyright 2019 Springer Nature Limited). Panel (C) Structures of major lipid-based nanocarriers—liposomes, solid lipid nanoparticles (SLNs), nanostructured lipid carriers (NLCs), and lipid nanoparticles (LNPs) illustrating their lipid architecture, encapsulated payloads, and roles as biocompatible delivery vehicles and Raman-reporting platforms for tracking lipid metabolism, nanoparticle fate, and cellular heterogeneity. The image was created in BioRender.com.

Historically, the scaffolds driving these systems have evolved significantly; liposomes, first described by Bangham in the 1960s,^65^ can encapsulate aqueous and hydrophobic payloads.^36,34^ Their development into PEGylated, long-circulating formulations enabled clinical translation, exemplified by Doxil, approved for ovarian cancer in 1999.^240–242^ While liposomes revolutionized drug delivery, their instability and limited retention led to SLNs. SLNs are submicron particles formed from lipids that remain solid at room and body temperature. SLNs have better stability and retention profiles.^39,56^ NLCs further improved lipid carriers by combining solid lipids and a fraction of liquid lipids, enhancing encapsulation efficiency and physicochemical stability.^74^ NLCs are considered second-generation LNPs and are produced by combining solid lipids with fractions of liquid lipid. This creates a less ordered matrix, improves drug loading, and can reduce drug expulsion during storage.^56^ Beyond therapeutics, lipid nanocarriers have also emerged as dynamic optical reporters for intracellular chemical imaging.^75^ Their soft, tunable membranes allow incorporation of Raman reporters for chemically specific imaging without toxic buildup.^76^ The combination of these structural and functional elements forms the basis of Raman-activated LNPs, as illustrated in Figure 8. Consequently, a standardized nomenclature is employed to describe these platforms based on their carrier composition, plasmonic integration, and reporter types. For instance, SLN- or NLC-based reporters track phase transitions and dynamic cargo deployment. LNP-Gold or hybrid lipid-metal nanohybrids signify the inclusion of metallic cores for SERS.^67–71^ Bioorthogonal LipoTags designate systems featuring narrow-line width functional groups such as alkynes, nitriles, deuterium tags, metal carbonyls, azo-BODIPY derivatives, Ru-polypyridyl, and porphyrin-metal conjugates, enabling multiplexed imaging in the cell-silent region, providing background-free tracking of metabolic reprogramming and NP fate.^243,244^

## Raman Reporters in Lipid NPs

Raman reporters are the molecular cores of lipid NPs, enabling chemically specific imaging, signal amplification, and multiplexed detection. When coupled with SERS (Figure 9), these reporters can be strategically positioned within the NP structure. They may be partitioned into the hydrophobic lipid layer, encapsulated inside the aqueous core, conjugated to the hydrophilic surface, or localized at the interface of a hybrid metal-lipid architecture. Their spatial localization directly governs localized electromagnetic enhancement factors, baseline colloidal stability, and excitation selectivity.^70,245,246,238^ Beyond static tracking, the incorporation of stimuli-responsive molecular linker enzymes, redox, or pH-activated signal generation. As a result, these systems facilitate real-time interrogation of dynamic cellular processes and microenvironmental shifts.^247,248^ Together, these rational design strategies position Raman-coded LNPs as powerful platforms for imaging-guided nanomedicines.

**Figure 9:**
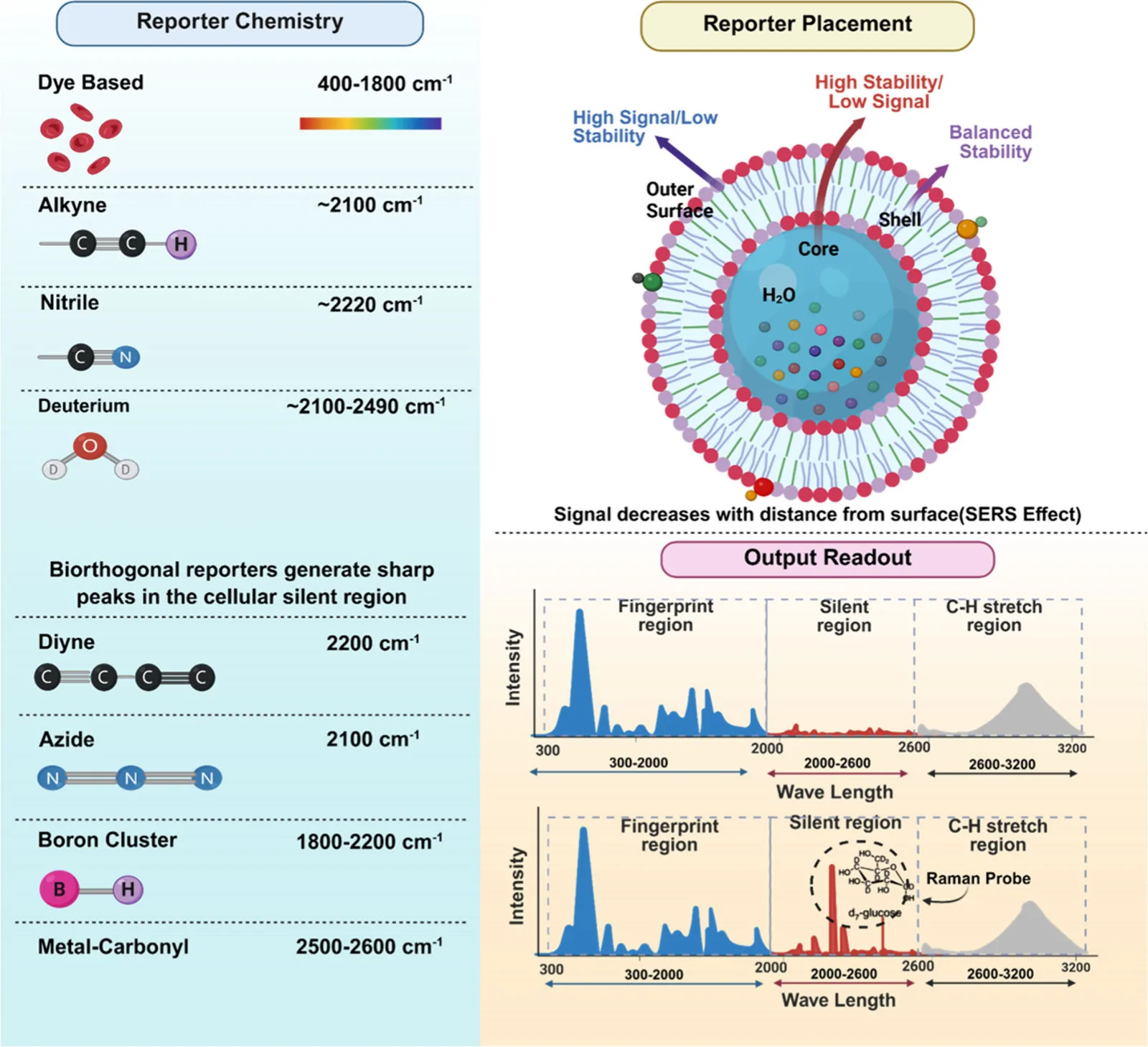
Schematic illustrating the influence of reporter chemistry and spatial localization on Raman signal generation in lipid nanoparticles (LNPs). Conventional dye-based reporters produce intense signals in the fingerprint region (400–1800 cm^–1^), whereas bioorthogonal reporters—including alkynes (∼2100 cm^–1^), nitriles (∼2220 cm^–1^), azides (∼2100 cm^–1^), diynes (∼2200 cm^–1^), deuterium labels (∼2100–2300 cm^–1^), boron clusters (1800–2200 cm^–1^), and metal–carbonyl probes (∼1900–2200 cm^–1^)—generate distinct peaks in the cell-silent region, enabling low-background, multiplexed detection. Reporter placement within the lipid bilayer, aqueous core, or nanoparticle surface modulates signal intensity, colloidal stability, and stimulus responsiveness. Together, reporter chemistry and localization determine the Raman spectral output and imaging performance. The image was created in BioRender.com.

## Bioorthogonal Raman Reporters vs Traditional Reporters

Raman reporters serve as molecular transducers, converting chemical or biological events into quantifiable vibrational signatures.^249^ Traditional dye-based reporters, such as organic chromophores, rely heavily on resonance Raman effects. When these molecules are excited near their electronic transitions, they yield very high signal enhancement factors exceeding 10^6^ −10^8^.^250,251^ However, these bulky traditional dyes often suffer from severe fluorescence background interference and photobleaching. They also experience photobleaching. Additionally, they can cause steric hindrance that may fundamentally alter the behavior of small biomolecules. To circumvent these limitations, bioorthogonal Raman reporters (BRRs) have emerged. These molecular probes operate in the cellular silent region, which is largely free of endogenous Raman vibrational signatures from cellular components.^243,70^ In BRRs, probes with the terminal/internal alkynes (R–C ≡C–H), nitriles (R-C≡N), and C–D bonds are typically used. They generate sharp, isolated peaks suitable for Raman spectroscopy and multiplexed imaging.^4,252^ Figure 9 summarizes the principles of BRRs, including silent-region reporters, isotopic encoding strategies, and their applications in metabolic imaging. These compact, chemically inert motifs integrated into metabolic precursors or drugs without perturbing native biochemical activity.^253^

Various architectures are possible for BRRs. Among those, alkynes exhibit the strongest Raman cross sections due to linear geometry and high polarizability anisotropy.^4,254^ Structural diversification from terminal to conjugated polyynes extends π-delocalization, tuning vibrational frequencies, and enabling spectral “color palettes” for multiplexed imaging.^255,256^ Isotopic substitution, especially ^13^C or ^2^H labeling, provides 40–80 cm^–1^ spectral shifts, expanding multiplexing channels from three to more than ten per cell.^46,45^ Nitriles provide moderate intensity but high environmental sensitivity, enabling ratiometric results of polarity, hydrogen bonding, and pH^257,258^ changes in the cells. Deuterium labeling, though weaker spectroscopically, offers unmatched biocompatibility for tracking lipid and protein turnover in real-time.^243^

From a functional perspective, dye-based reporters are optimal for maximal intensity assays, whereas bioorthogonal reporters excel in live-cell imaging, metabolic flux tracking, and low-background applications.^4^ Modern workflows integrate both classes, using dyes for initial high-signal screening and BRRs for mechanistic follow-up studies.^259^ This hybrid approach enables precise molecular design tailored to specific biological questions without forcing universal reporter solutions. Emerging strategies include multiplexed silent region reporters, organelle-targeted BRRs, and AI-assisted spectral deconvolution. These advances further enhance sensitivity, interpretability, and temporal resolution.^249^ Ultimately, bioorthogonal Raman reporters embedded in lipid or NP scaffolds provide real-time chemical-metabolic imaging in living cells. These help bridge molecular specificity with translational relevance.^4,63^

## Reporter Placement Strategies in Lipid Nanoparticles

Hydrophobic reporters spontaneously partition into the lipid bilayer through acyl-chain compatibility during vesicle self-assembly.^75,246^ This approach achieves high reporter loading with minimal synthetic complexity and preserved colloidal stability.^260^ For example, in the hybrid “LipoGold” architectures, bilayer-embedded reporters align precisely at the interface to couple with clustered gold NPs forming plasmonic hotspots.^70,261^ Within these soft matrices, membrane stiffness and lipid polymorphism can be exploited to regulate NP clustering, directly controlling hotspot density and signal uniformity in real time. Liquid-crystalline membranes enhance signal reproducibility; gel-phase lipids restrict reporter diffusion and reduce enhancement.^262^ NP size affects membrane integrity, with ultrasmall particles integrating without vesicle destabilization.^263^ Core encapsulation sequesters reporters within aqueous or lipidic interiors, providing chemical protection.^264,238^ Core-loaded reporters resist enzymatic degradation and enable ratio metric internal sensing channels.^265,266^ However, electromagnetic field decay exponentially from the core to the surface of the particles, reducing the enhancement achieved for core-loaded reporters compared to surface-based attachment.^261,267^ Surface-anchored reporters maximize plasmon proximity, yielding strong and reproducible SERS signals.^247^ Thiolate reporters form stable metal–sulfur bonds, ensuring defined orientation and spectral reproducibility.^123,268^

Multilayer reporter architecture enables additive electromagnetic coupling, approaching single-molecule sensitivity.^269^ Excessive surface crowding can impair uptake, necessitating precise optimization of reporter density.^270,271^ SLNPs entrap reporters within crystalline matrices that provide mechanical stability and controlled release.^272,273^ Nanostructured lipid carriers improve reporter loading by disrupting lipid crystallinity and preventing reporter expulsion.^274^ Lipid polymorphism alters vibrational band positions, enabling real-time monitoring of matrix order.^275^ Membrane stiffness regulates NP clustering, directly controlling hotspot density and signal uniformity.^276^ Redox-active lipids enable *in situ* metal growth within vesicles for controlled hybrid assembly.^277,278^ Collectively, reporter placement represents a primary design axis linking NP architecture with optical performance and biological fidelity.^279^

## Smart Linkers and Enzyme-Activated Raman Reporters

Conventional Raman reporters generate static vibrational signals that primarily indicate molecular presence rather than biochemical activity. Smart linker strategies address this limitation by introducing stimulus-responsive chemistries that convert specific biochemical events into “signal-on” Raman responses, enabling spatially and temporally resolved imaging of pathological microenvironments such as tumors and inflamed tissues.^280^ These systems integrate molecular recognition with optical amplification, advancing Raman imaging toward activity-encoded molecular sensing. A typical smart-linker design comprises three modular components: (i) a vibrational distinct Raman reporter; (ii) a biologically cleavable or transformable substrate responsive to enzymatic, redox, or pH triggers; and (iii) a linker scaffold anchoring the construct to a lipid or NP carrier.^281^ Biochemical activation induces cleavage or chemical transformation of the probe. As a result, molecular aggregation, electronic resonance, or proximity to plasmonic surfaces is altered, switching the Raman signals from an “off” to an “on” state within highly spatial region.^123^

Enzyme-activated Raman probes (EARPs) represent the most developed class of smart-linker systems. EARPs exploit substrate-enzyme recognition to translate catalytic turnover directly into vibrational contrast. This enables activity-based imaging of proteases, glycosidases, and peptidases associated with cancer and inflammation.^282,45^ For example, 9CN- rodhol platform exemplifies the approach; enzymatic deprotection restores π-conjugation and induces molecular nanoaggregation, and markedly enhances SRS.^248^ This activation increases local reporter concentration while limiting diffusion, yielding high signal-to-background ratios suitable for precise tumor-margin detection.^283^ At the mechanistic level, most EARPs amplify signals via aggregation-induced enhancement or resonance restoration. In these, enzymatic cleavage couples’ absorption with Raman scattering.^248^ Quantitative metrics, including Raman gain, turnover number, and signal-to-background, enable objective comparison of smart-linker efficiency.^284^

Enzyme-responsive linker chemistry governs specificity, kinetics, and biostability. Peptide-based linkers like Pro-Leu-Gly enable matrix metalloproteinase (MMP)-triggered Raman signals.^244^ β-glucosidase-cleavable glycosidic linkers liberate nitrile reporters upon hydrolysis. Under optimized SERS conditions, these systems achieve detection limits in the nM to pM.^285^ Redox- and pH-responsive linkers such as hydrazone or acetal enable selective intracellular cleavage, minimizing extracellular background activation.^286,287^ Importantly, linker cleavage kinetics must be matched to enzyme expression levels and intracellular residence times to preserve temporal fidelity while suppressing nonspecific activation.^284,288^ Encapsulation within LNPs further improves colloidal stability and prolongs circulation. It also shields inactive reporters from nonspecific cleavage, thereby reducing false-positive activation in complex biological environments.^289^

Beyond single-enzyme sensing, isotopic editing has enabled multiplexed biochemical imaging with chemically identical probes that exhibit spectrally distinct vibrational signatures. Isotopically tuned 9CN-rhodol derivatives (^12^C^14^N, ^13^C^14^N, and ^13^C^15^N) generate resolvable Raman peaks separated by 30–90 cm^–1^ within the silent region, permitting simultaneous visualization of multiple enzyme activities under SRS microscopy.^282,45^ Because isotopic substitution preserves molecular structure, quantitative cross-enzyme comparisons can be performed without perturbing biological function.^256^ Recent developments use logic-gated, multistimuli probes, where dual MMP-2/cathepsin B substrates activate Raman signals selectively in tumors.^290^ Incorporation of additional redox- or pH-responsive elements enables higher-order logic operations that better reflect cellular signal integration.^291^

## Raman Stable Isotopes Probes

Multicolor Raman coding enables monitoring of multiple biomolecules with high spectral fidelity, surpassing the limits of detection of fluorescence modalities. This is possible due to narrow vibrational line widths of Raman peaks (≤1 cm^–1^ FWHM).^6^ To build dense molecular color palettes, encoding strategies exploit the cellular silent region, a frequency window entirely free from the background vibrations of endogenous cellular components.

Isotopic editing shifts vibrational frequencies predictably via the mass-frequency relationship derived from a classical two-mass spring model

$$V=\frac{1}{2\pi c}\sqrt{\frac{k}{\mu }}$$

Here, V represents the vibrational wavenumber, *k* represents the bond force constant, and c denotes the speed of light.

$$\mu =\frac{m_{1}m_{2}}{m_{1}+m_{2}}$$

The vibrational frequency is inversely proportional to the square root of the reduced mass. Substitution with heavier isotopes increases μ and consequently decreases the vibrational frequency. Because isotopic substitution increases μ without altering the bond’s native force constant *k*, it modifies the spectral frequency without changing the probe’s chemical profile or biological processing kinetics.^292^ For example, systematic Carbon-13 substitution in alkyne tags produces cleanly resolved peaks ^12^C≡^12^C (2125 cm^–1^), ^13^C≡^12^C (2077 cm^–1^), and ^13^C≡^13^C (2048 cm^–1^),^46^ ^15^N incorporation in nitriles red-shifts frequencies to ≈2200 cm^–1,^ ^248^ (Figure 10). Combining isotopic tuning enables >10 distinct channels within a single excitation window, maintaining consistent uptake and metabolism relatively unaltered.^256^

**Figure 10:**
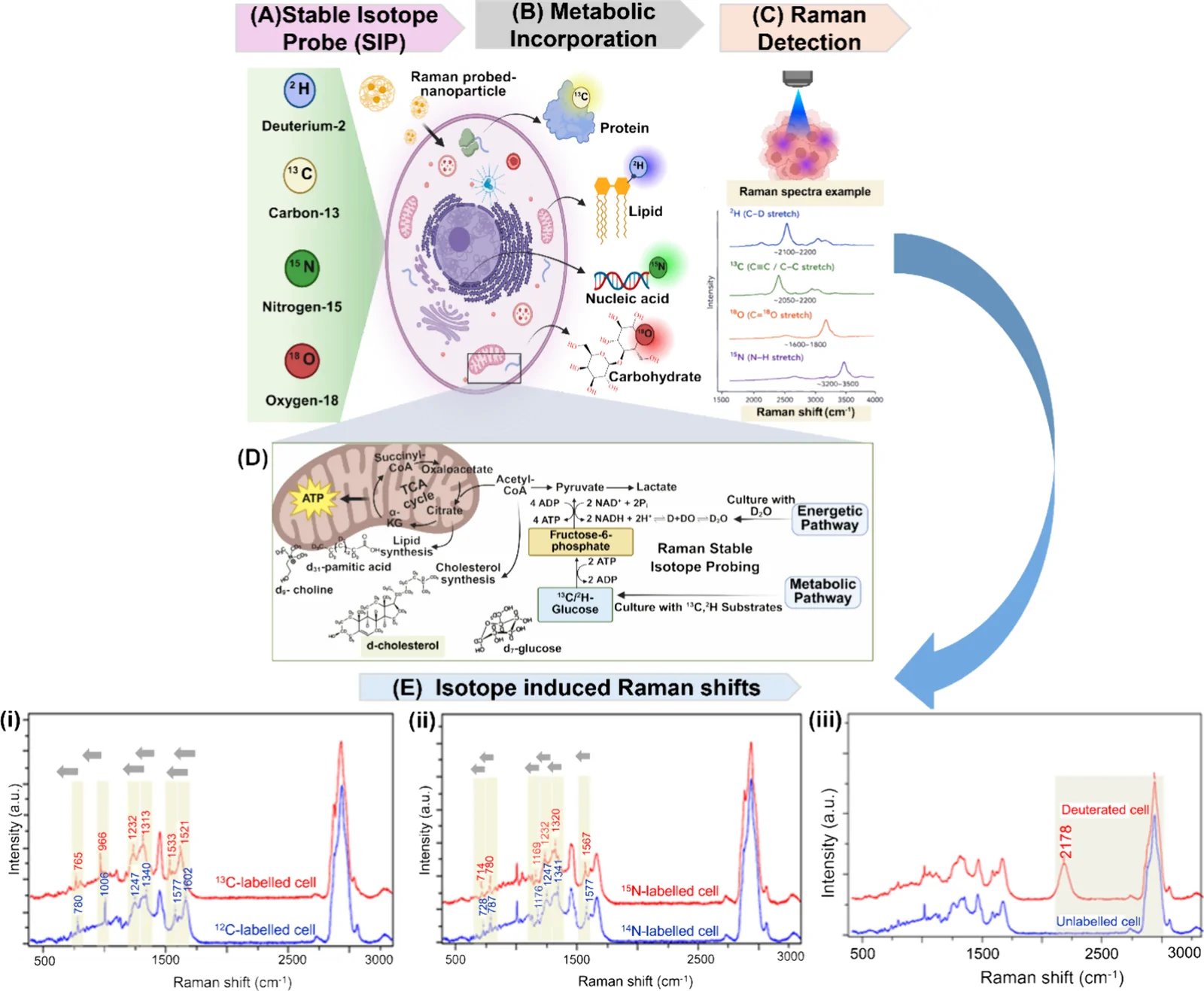
Schematic workflow and metabolic mechanisms of Raman-coupled stable isotope probing (Raman SIP). (A) Selection of stable isotope tracers, including ^2^H, ^13^C, ^15^N, and ^18^O. (B, D) Cellular uptake and metabolic incorporation of isotopes into biomolecules through glycolysis, the TCA cycle, lipogenesis, and biosynthetic pathways, generating labeled lipids, proteins, nucleic acids, carbohydrates, and metabolites (e.g., d_31_-palmitic acid, *d*_9_-choline, and *d*_6_-cholesterol). (C) Raman detection of isotope incorporation, where increased bond mass induces characteristic red shifts to lower wavenumbers, including C–D stretching (∼2040–2300 cm^–1^) and isotope-dependent shifts in C=C/C–C, ^18^O-containing, and ^15^N-containing vibrations. (E) Representative single-cell Raman spectra comparing unlabeled and isotope-labeled cells, illustrating ^13^C- and ^15^N-induced fingerprint-region shifts (700–1700 cm^–1^) and the emergence of a distinct C–D band in the cell-silent region (∼2178 cm^–1^), enabling background-free monitoring of *de novo* biosynthesis and metabolic activity. Panels A–E adapted with permission from ref 293 (Copyright 2016 Elsevier Ltd.). The schematic elements were created with BioRender.com.

## SERS Probes

The integration of plasmonic nanostructures into lipid-based carriers creates multifunctional hybrids for imaging, sensing, and therapy.^294^ Lipid bilayers provide biocompatible matrices that stabilize noble metals (Au, Ag, and alloys). This enables localized surface plasmon resonance (LSPR) for enhanced electromagnetic fields, single-particle tracking, and real-time molecular detection.^295,296,261^ Lipid-metal synergy tunes dielectric properties, confines fields, and regulates protein corona formation and cellular uptake.^295,297^

## Plasmonic Enhancement of Raman Signals

Surface-enhanced Raman scattering (SERS) exploits the plasmonic properties of noble metals to amplify inherently weak Raman signals.^298^ Nanostructures of gold (Au), silver (Ag), and copper (Cu) support localized surface plasmon resonances (LSPR), which is a phenomenon where incident light drives the coherent oscillation of conduction-band electrons. This collective oscillation produces intense, localized electromagnetic fields known as “hot spots” that amplify Raman scattering by factors up to ∼ 10^6^–10^8^ or more.^299^ The strength and spectral position of plasmonic enhancement depend critically on nanoparticle geometry, size, composition, interparticle spacing, and dielectric environment.^300,301,43^ Anisotropic structures such as nanorods, stars, and core–shell nanostructures generate high field enhancements via sharp features and coupled plasmon modes, enabling improved hotspot density for ultrasensitive detection (Figure 11).^302,303^ Hybrid plasmonic designs further boost performance; for example, combining Au with two-dimensional materials such as WS_2_, yields synergistic electromagnetic and chemical enhancement, with reported enhancement factors up to ∼10^9^ and practical detection of bacterial analytes at ∼10^4^ CFU mL^–1^.^304^

**Figure 11:**
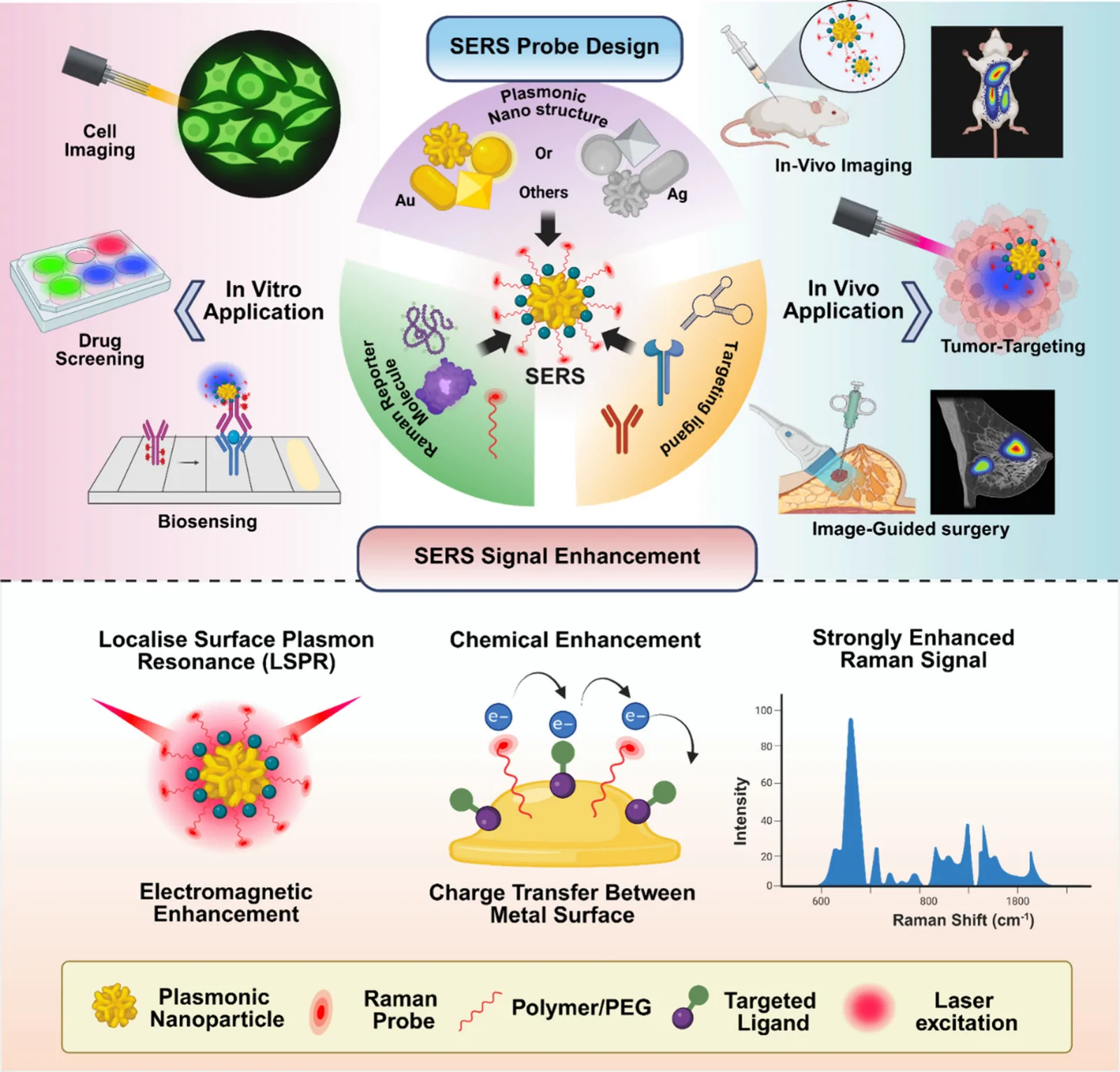
Overview of surface-enhanced Raman scattering (SERS) probe design, signal enhancement, and biomedical applications. The schematic illustrates a typical SERS probe comprising a plasmonic nanostructure (e.g., Au or Ag), Raman reporter molecules, target-specific ligands, and a polymer/PEG coating. Upon laser excitation, Raman signals are amplified through localized surface plasmon resonance (LSPR)-mediated electromagnetic enhancement and charge transfer–based chemical enhancement. These engineered probes enable diverse *in vitro* applications, including single-cell imaging, high-throughput drug screening, and microfluidic biosensing, as well as *in vivo* applications such as whole-body imaging, tumor targeting, and Raman-guided surgery. The schematic image was created in BioRender.com.

Electromagnetic effects dominate SERS, while charge-transfer interactions add chemical amplification.^305^ Rational engineering of surface chemistry and ligand interactions can maximize both electromagnetic and chemical enhancement in a single platform.^306^ Beyond single-molecule sensitivity, plasmonic SERS substrates have been integrated into biomedical imaging and sensing workflows.^307^ Targeted Au and Ag nanoparticles conjugated to biomolecule ligands or antibodies enable multiplexed detection of cancer biomarkers in tissues, offering high spatial resolution and chemical specificity.^308^ Similar platforms have been facilitating chemical sensing, where plasmonic field enhancement and computational spectral analysis facilitate the resolution of complex biochemical signatures in neuronal environments.^309^

Emerging designs include magnetically coupled plasmonic NPs. These systems combine analyte enrichment with the SERS detection. Other designs use hybrid photothermal SERS agents that integrate diagnostic and therapeutic functions.^310^ Alternative plasmonic materials such as aluminum and copper, previously limited by oxidation, show promise when engineered with protective coatings, offering cost-effective, scalable SERS substrates for chemical sensing.^311^ Thus, through careful control of the nanostructure design and surface chemistry, plasmonic metals convert weak Raman scattering into a quantitative, high-contrast spectroscopic signal. This significantly enhances the sensitivity and reliability of the Raman-based measurements. As a result, plasmonic metals support advanced biosensing, high-resolution imaging, and trace detection across biological and chemical systems.^298^

## Comparative Maturity of Raman Modality/Probe Combinations for Live-Cell, Deep-Tissue, Multiplexed, and Quantitative Metabolic Readouts

Raman-based metabolic imaging platforms differ substantially in maturity (Table 4) depending on the intended readout. The most mature combinations are not necessarily the most sensitive; rather, they are those with demonstrated live-cell compatibility, quantitative robustness, tissue penetration, multiplexing capacity, and reproducibility across biological systems.

**Table 4:** Comparison of Different Raman Modalities for Metabolic Imaging

Modality/probe combination	Live-cell capability	Deep-tissue imaging	Multiplexing	Quantitative metabolic readout	Performance ranges	Best-suited application	Key limitation
Label-free spontaneous Raman	Moderate	Low–Moderate	High spectral content	High chemical specificity; moderate throughput	Broad spectral range: ∼400–3200 cm^–1^; spatial resolution ∼0.3–1 μm; acquisition commonly seconds per spectrum	Full-spectrum metabolic fingerprinting of cells/tissues	Slow imaging; weak Raman signal
Label-free SRS	Very high	High	Moderate	High	Pixel dwell times often μs-scale; commonly targets CH_2_/CH_3_ bands near ∼2845–2930 cm^–1^; subcellular resolution	Fast live-cell lipid/protein imaging, lipid droplets, tissue metabolism	Usually limited spectral coverage unless hyperspectral SRS is used
D_2_O-SRS/DO-SRS	Very high	High	Moderate	Very high	C–D signal in ∼2040–2300 cm^–1^ Raman-silent region; enables quantitative biosynthetic activity imaging in living cells and animals	Metabolic flux, protein/lipid synthesis, tumor metabolism, antimicrobial response	Requires isotope exposure; reports turnover rather than full biochemical composition
Alkyne-tag SRS	Very high	Moderate–high	High	High	Alkyne peaks usually ∼2100–2300 cm^–1^; reported SRS detection limit for alkynes down to ∼200 μM with 100 μs acquisition in EdU imaging	Live-cell tracking of small biomolecules, lipids, nucleotides, drugs	Requires exogenous tagged analogs; biological perturbation must be controlled
CARS label-free imaging	High	High	Low–moderate	Moderate	Fast, nonlinear Raman imaging; strong for lipid-rich structures and myelin	Lipid-rich tissues, live-cell lipid droplets, myelin imaging	Nonresonant background complicates weak-band detection and quantification
Multiplex CARS/hyperspectral CARS	Moderate	Moderate	Moderate–high	Moderate–high	Fingerprint chemical imaging demonstrated, but instrumentation and analysis are more complex	Spectrally resolved tissue imaging	Less widely adopted than SRS; quantitative analysis is harder
SERS nanoparticles/Raman nanotags	Moderate	Moderate	Very high	Low–moderate	Raman nanotags can be multiplexed; up to 10 SERS nanoparticle flavors were separated *in vivo* in a landmark study	Multiplexed cell/tissue labeling, biodistribution, targeted detection	Signal depends on nanoparticle localization and hot spots; less reliable for metabolic flux
Lipid-coated SERS nanoparticles	Moderate	Moderate	High	Low–moderate	Very high sensitivity; useful for tracking nanoparticle fate or local biomarkers	Targeted imaging, membrane interaction, nanoparticle tracking	Usually reports the reporter/nanoparticle, not endogenous metabolism
Isotope labeling + spontaneous Raman	Moderate	Low	Moderate	High chemical specificity; low throughput	C–D and isotope-shifted bands can be detected, but acquisition is slow	Single-cell metabolic phenotyping, microbial metabolism	Too slow for large live-cell or deep-tissue imaging
Lipid nanoparticles/SLNs/NLCs with Raman reporters	Moderate	Moderate	Moderate–high if reporter encoded	Moderate for tracking; low for flux	Best for carrier uptake and biodistribution rather than direct metabolic activity	Nanoparticle delivery, uptake, lipid handling	Metabolic interpretation is indirect unless reporter is metabolically incorporated

Overall, label-free SRS and isotope-assisted SRS are the most mature platforms for live-cell metabolic imaging (Figure 12) because they combine fast acquisition, subcellular spatial resolution, and compatibility with living cells and tissues. SRS has become a high-speed chemical imaging method for living systems,^312^ while D_2_O-SRS directly links Raman contrast to biosynthetic activity through enzymatic incorporation of deuterium into C–D bonds in newly synthesized biomolecules.^313^ The C–D signal appears in the Raman-silent region, reducing endogenous background and enabling quantitative metabolic readouts in cells, tissues, and animal models. Therefore, for dynamic metabolism, lipid synthesis, protein synthesis, and treatment-response studies, D_2_O-SRS and alkyne-tag SRS are currently the strongest modality/probe combinations.^164,46^

**Figure 12:**
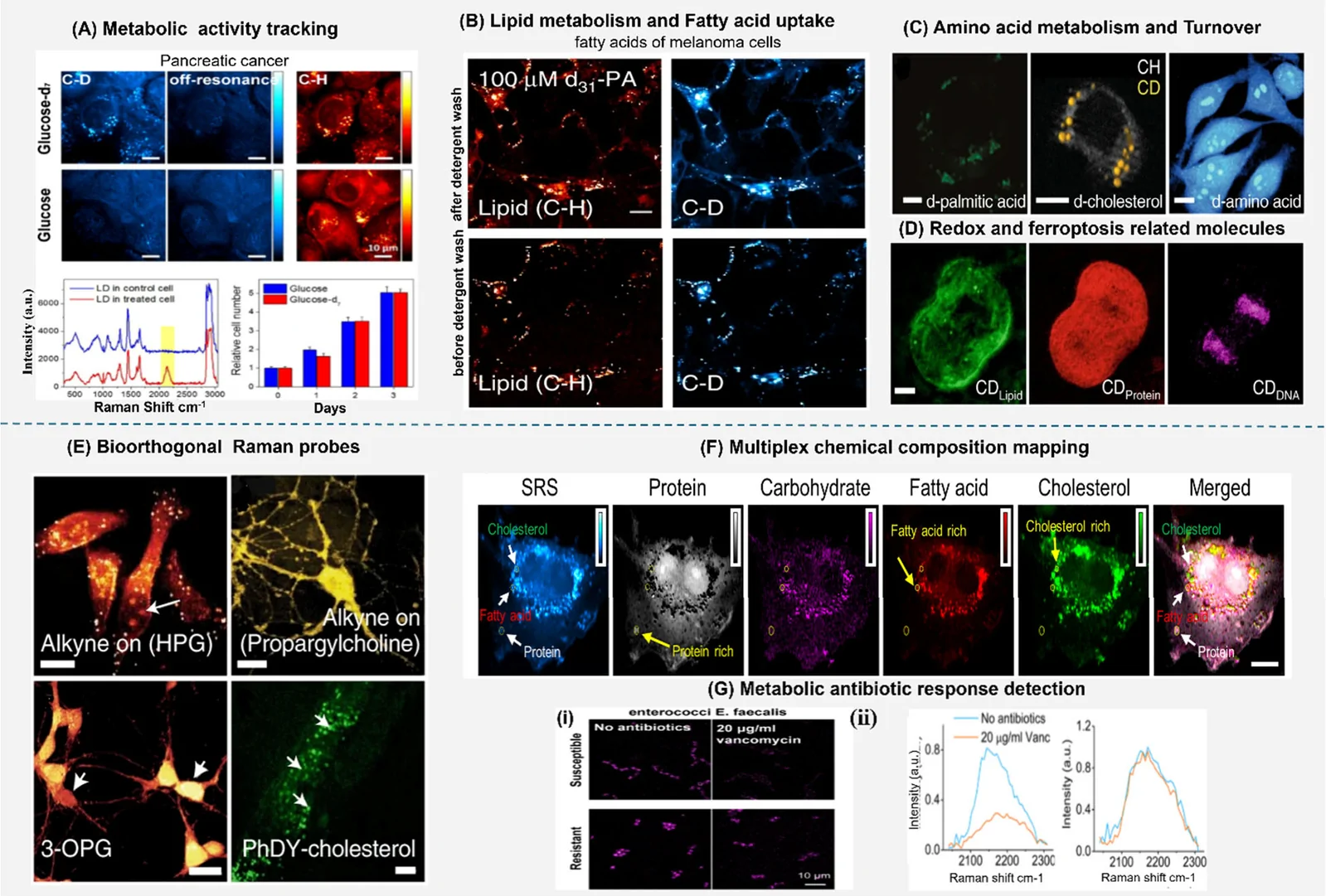
Representative applications of stable isotope probe (SIP)-based stimulated Raman scattering (SRS) imaging. (A) Glucose-d_7_ labeling visualized through C–D and C–H channels, with minimal cytotoxicity confirmed by MTT assay. (B) SRS imaging of d_31_-palmitic acid showing incorporated and soluble lipid pools before and after washing (scale bar: 20 μm). (C) Visualization of d_31_-palmitic acid incorporation into lipid droplets and membranes, d_38_-cholesterol uptake and storage, and deuterated amino acid incorporation into newly synthesized proteins (scale bars: 5–10 μm). (D) D_2_O-based labeling of newly synthesized proteins, lipids, and DNA for global metabolic imaging in live cells (scale bar: 10 μm). (E) Multiplex alkyne-tagged SRS imaging of proteins (HPG), choline (propargylcholine), glucose (3-OPG), and cholesterol (PhDY-cholesterol) in cells and *C*. *elegans*. (F) Hyperspectral SRS (hSRS and h^2^SRS) mapping of proteins, carbohydrates, fatty acids, cholesterol, and nucleic acids in cholesterol-enriched U87 cells. (G) Detection of vancomycin susceptibility in enterococci by SRS imaging at the C–D band (2178 cm^–1^) and corresponding spectra of susceptible (VSE) and resistant (VRE) bacteria following antibiotic treatment. Panels A and G adapted with permission from ref 317 (Copyright 2025 American Chemical Society, Available under CC BY-NC-ND 4.0 License). Panel B Reproduced from ref 318 (Copyright 2020 Springer Nature). Available under CC by 4.0 License. Panels D and E adapted with permission from ref 195 (Copyright 2019 Springer Nature Limited). Panel F reproduced from ref 319 (Copyright 2023 American Association for the advancement of science). Available under CCBY-NC 4.0 License.

For deep-tissue imaging, coherent Raman methods, especially SRS and CARS, are more mature than spontaneous Raman because their nonlinear signal generation and laser scanning implementations support faster imaging in thick tissues. CARS has been widely used for lipid-rich biological structures such as myelin and lipid droplets, but its nonresonant background complicates quantitative interpretation.^314^ SRS is generally preferred for quantitative chemical imaging because the SRS signal is linearly proportional to molecular concentration under appropriate conditions, whereas CARS intensity has a more complex relationship to concentration.^312^

For multiplexed readouts, SERS nanotags and Raman-silent bioorthogonal probes are the most mature. SERS nanoparticles can provide highly multiplexed optical barcodes because Raman peaks are narrow and chemically tunable; a landmark *in vivo* study separated up to 10 different SERS nanoparticle signatures.^315^ However, SERS is better suited for multiplexed labeling, biodistribution, and targeted molecular detection than for endogenous metabolic flux because signal intensity depends on nanoparticle distribution, aggregation state, and local plasmonic hot spots. In contrast, alkyne, nitrile, and deuterium Raman tags provide metabolically interpretable multiplexing when the tag is incorporated into defined biomolecular pathways.^46^

For quantitative metabolic readouts, D_2_O-SRS and isotope/bioorthogonal SRS represent the strongest approaches, followed by label-free SRS and spontaneous Raman, whereas SERS-based approaches are generally less robust for absolute quantification.^164,316^ Spontaneous Raman remains highly valuable, but its strength is different: it provides the broadest chemical specificity and full-spectrum metabolic fingerprinting rather than speed. Thus, spontaneous Raman is best positioned as an unbiased metabolic phenotyping platform, whereas SRS-based methods are best positioned for fast, live cell, and quantitative dynamic imaging.

## Raman-Metabolic Imaging: Obstacles and Opportunities

Even having exceptional chemical specificity and label-free capability, Raman-based metabolic imaging physical, instrumental, computational, and translational barriers that limit its widespread biomedical application. Quantum electrodynamics predicts an extremely small Raman scattering cross sections (σ_Raman_, typically 10^–30^ to 10^–28^ cm^2^), several orders of magnitude lower than molecular absorption cross sections (10^–16^ to 10^–15^ cm^2^).^320^ This happens because spontaneous Raman involves vacuum-field-induced spontaneous emission absent in absorption.^321^ Consequently, spontaneous Raman requires prolonged integration times of seconds to minutes per pixel, limiting live-cell imaging and high-throughput analysis. Although CRS enhances signals by up to 5 orders of magnitude, sensitivity remains insufficient in the spectrally crowded fingerprint region, where overlapping lipids, proteins, and nucleic acids requires multivariate analysis (e.g., PCA, PLS-DA, MCR-ALS).^322–325^ However, MCR-ALS, depends strongly on user-defined parameters, and many classification approaches distinguish mathematical rather than chemical variations, increasing the risk of overinterpretations.^326,327^ CRS also exhibits an inherent trade-off between spectral bandwidth, imaging speed, and image fidelity. Hyperspectral SRS acquires spectra within microseconds but over only 200 cm^–1^. Alternatively, ultrafast SRS reaches frame rates of 2 kHz using only four vibrational frequencies, whereas broadband CARS spans ∼3000 cm^–1^ but requires longer pixel dwell times (∼3.5 ms). Increasing laser power to compensate for weak Raman signals causes photothermal heating, oxidative damage, and phototoxicity, producing myelin splitting, shockwave formation, and membrane blebbing in CARS.^328^ While SRS sensitivity remains constrained by photodamage despite operation near the shot-noise limit.^329^ Conventional optical Raman imaging is limited to depths of ∼20–100 μm in highly scattering tissues like the brain and ∼300–500 μm in less scattering tissues. Although tissue clearing extends imaging depth to ∼1 mm, it is incompatible with live imaging and can cause biomolecular loss, particularly of lipids.^322,330^ But these methods are incompatible with living samples and cause biomolecular loss, particularly cellular lipids.^331^

NP-enhanced Raman imaging introduces additional translational barriers. Although Au- or Ag-based SERS substrates offer superb sensitivity, they often accumulate in the liver and spleen with pharmacokinetics governed by particle size, morphology, and surface chemistry.^332,333^ Incomplete clearance and prolonged tissue retention raise concerns regarding chronic toxicity and off-target biological effects, particularly for nonbiodegradable nanomaterials.^334,332^ Clinical translation is further hindered by stringent regulatory requirements for both NP probes and imaging platforms, despite several Au NP formulations entering clinical evaluation.^335,336^ Clinical Raman imaging also remains limited by shallow penetration, relatively slow acquisition, and reliance on specialized fiber-optic, endoscopic applications that demonstrate its clinical feasibility,^337,176^ Finally, the lack of standardized preprocessing and multicenter spectral databases could delay clinical implementation.^335,336,338^ Future advances will depend on biodegradable or renally clearable Raman probes, improved clinical Raman instrumentation, standardized imaging workflows, and harmonized regulatory pathways to enable safe and reproducible implementation in healthcare settings.^332,327^

### Metabolic Imaging Applications

#### In Vitro

Lipid-Raman probes provide a powerful and minimally perturbative platform for the direct visualization and quantitative analysis of intracellular lipid metabolism at single-cell resolution. These probes generate vibrational fingerprints of lipid chain length, unsaturation, and packing density, enabling *in situ* lipid mapping without perturbing cellular metabolism.^4^ Unlike fluorescence reporters, Raman-active lipid probes exploit intrinsic or bioorthogonal vibrational signatures for precise, label-free metabolic readouts.^339^ Among these strategies, bioorthogonal alkyne-tag Raman imaging enables robust tracking of fatty-acid uptake, esterification, and intracellular redistribution without disrupting lipid flux.^340^ Detection in the Raman cell-silent window (∼2100–2300 cm^–1^) enables selective, quantitative assessment of exogenous lipid utilization and remodeling. Complementarily, Raman band ratios associated with C=C stretching modes encode lipid unsaturation levels, providing a direct readout of lipid peroxidation during oxidative stress or ferroptotic signaling.^243,341^

Not all the peaks in a lipid Raman spectrum exhibit equal intensity. Coherent Raman modalities such as SRS can selectively enhance specific bond vibrations in real time, although with a limited dynamic wavenumber range. It has been used for imaging of lipid uptake, trafficking, and storage at subsecond temporal resolution.^347^ Hyperspectral SRS with chemometrics has been used to distinguish multiple fatty acid species, revealing lipid composition changes such as increased saturation in drug-induced steatosis in hepatocellular carcinoma models.^348^ Recently, lipid-based Raman reporters have been utilized to track lipid droplet (LD) biogenesis, capturing early nucleation and revealing intracellular lipid storage pathways critical for energy homeostasis and disease progression.^347^ These capabilities are further extended through hybrid lipid–plasmonic architectures, such as LipoGold constructs (Figure 13). These systems exploit localized electromagnetic field enhancement to enable metabolic imaging within subcellular organelles. Such organelles are otherwise inaccessible to spontaneous Raman microscopy.^70^ In another example, lipid-based Raman probes were used for investigating lipid heterogeneity and spatially map different metabolic zones in a 3D tumor spheroid model, highlighting differences between hypoxic cores and proliferative peripheries.^349^ Raman-based metabolic imaging inherently provides direct quantitative and photostable measurements. This is because the Raman signal reflects the molecular composition of the sample. In contrast, fluorescence imaging depends on the intensity of dye emission. When combined with computational spectral analysis, Raman methods are capable of profiling single-cell lipid metabolic heterogeneity and adaptive remodeling in health and disease.^243,350^ In summary, Raman-active lipid probes including alkyne-tagged, deuterated, and hybrid lipid–metal nanostructures, offer chemically optimized, subcellular, and quantitative insights into lipid metabolism, dynamics, and disease-associated remodeling beyond conventional imaging.

**Figure 13:**
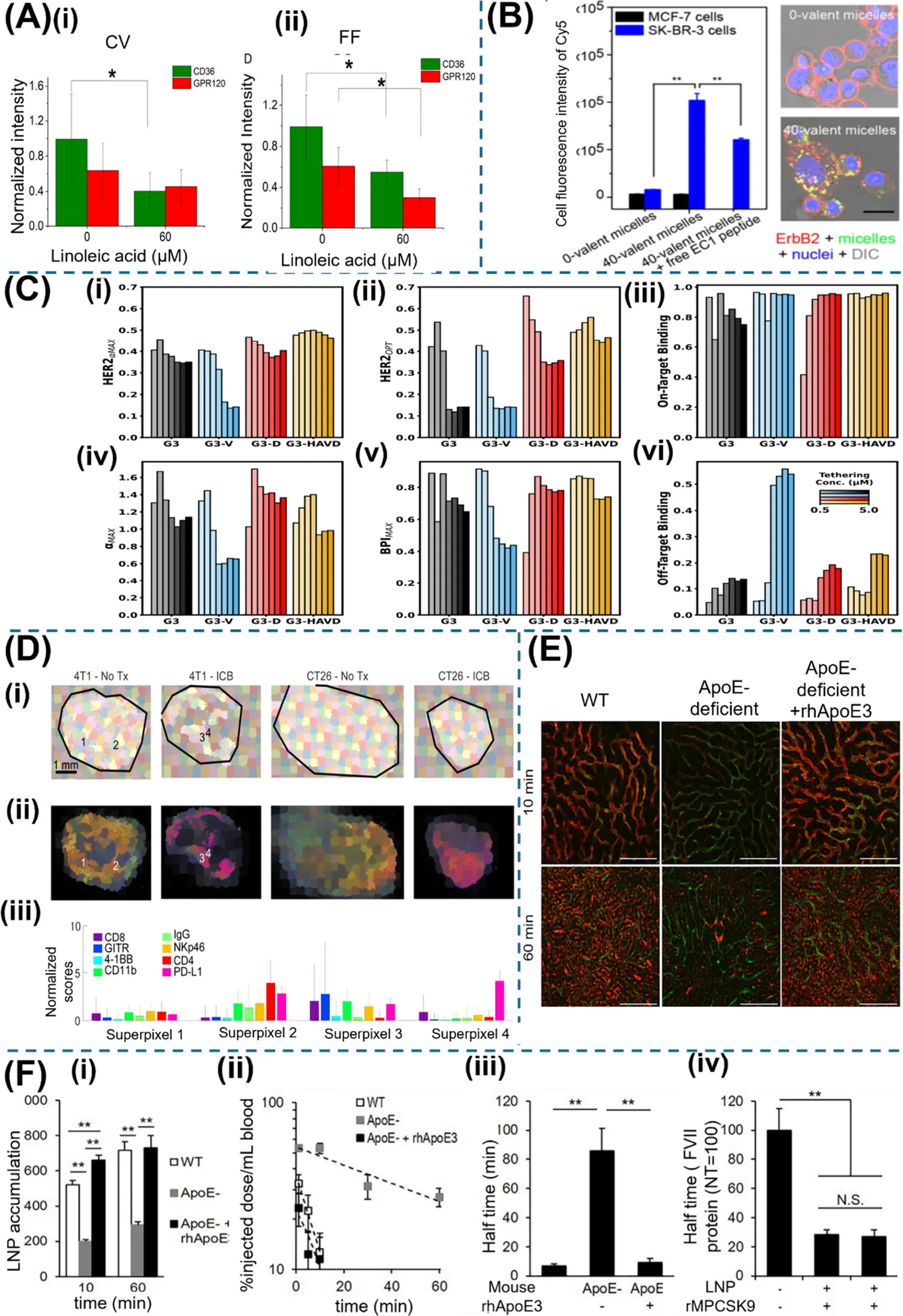
Representative applications of SERS and lipid nanoparticle (LNP) platforms in biomedical imaging and targeted delivery. (A) SERS intensity in primary circumvallates (CV) and foliate (FF) papillae cells with or without linoleic acid (LA) treatment (60 μM; *P* < 0.05; scale bar: 10 μm). (B) Enhanced ErbB2-dependent uptake of EC1-functionalized micelles in SK-BR-3 cells demonstrated by flow cytometry and confocal imaging, with inhibition by free EC1 (*P* < 0.01; scale bar: 12 μm). (C) HER2 binding analysis of DARPin constructs showing valency- and affinity-dependent targeting and increased off-target binding of G3-V at high tethering densities. (D) Tumor segmentation by superpixel clustering based on Raman nanoprobe uptake profiles for spatial classification of tumor regions. (E) ApoE-dependent blood clearance and hepatic accumulation of bare LNPs in wild-type and ApoE-deficient mice, with liver localization shown by histology (scale bar: 100 μm). (F) Quantification of intrahepatic LNP accumulation, blood pharmacokinetics, circulation half-life, and LDLR-dependent gene silencing following rmPCSK9 pretreatment (*P* < 0.05, *P* < 0.01). Panel A adapted with permission from ref 342 (Copyright 2020 National Academy of Sciences). Panel B reproduced from ref 343 (Copyright 2026 MDPI). Available under CC BY 4.0 License. Panels C reproduced from ref 344 (Copyright 2025 Taylor & Francis). Available under CC BY-NC 4.0 License. Panel D adapted from ref 345 (Copyright 2022 Royal Society of Chemistry). Panels E and F adapted with permission from ref 346 (Copyright 2020 Elsevier B.V.).

#### *In Vivo* Applications of Raman Reporters

The translation of lipid Raman nanoprobes from *in vitro* systems to living organisms offers unprecedented opportunities to interrogate and quantify metabolic and molecular processes in real time. This approach enables a deeper understanding of dynamic biological events. *In vivo* applications leverage the chemical specificity of Raman-active probes **(** Figure 14 **)**, the multiplexing capabilities of vibrational tags, and advanced imaging modalities to monitor dynamic biological phenomena with spatial and temporal resolution.^351^ By integrating tumor and metabolic imaging, multiplexed biomarker detection, and strategies for optimizing imaging depth and signal-to-noise, *in vivo* studies will enable quantitative assessment of lipid metabolism, protein turnover, oxidative stress, and therapeutic responses within native tissue.^352,164^

**Figure 14:**
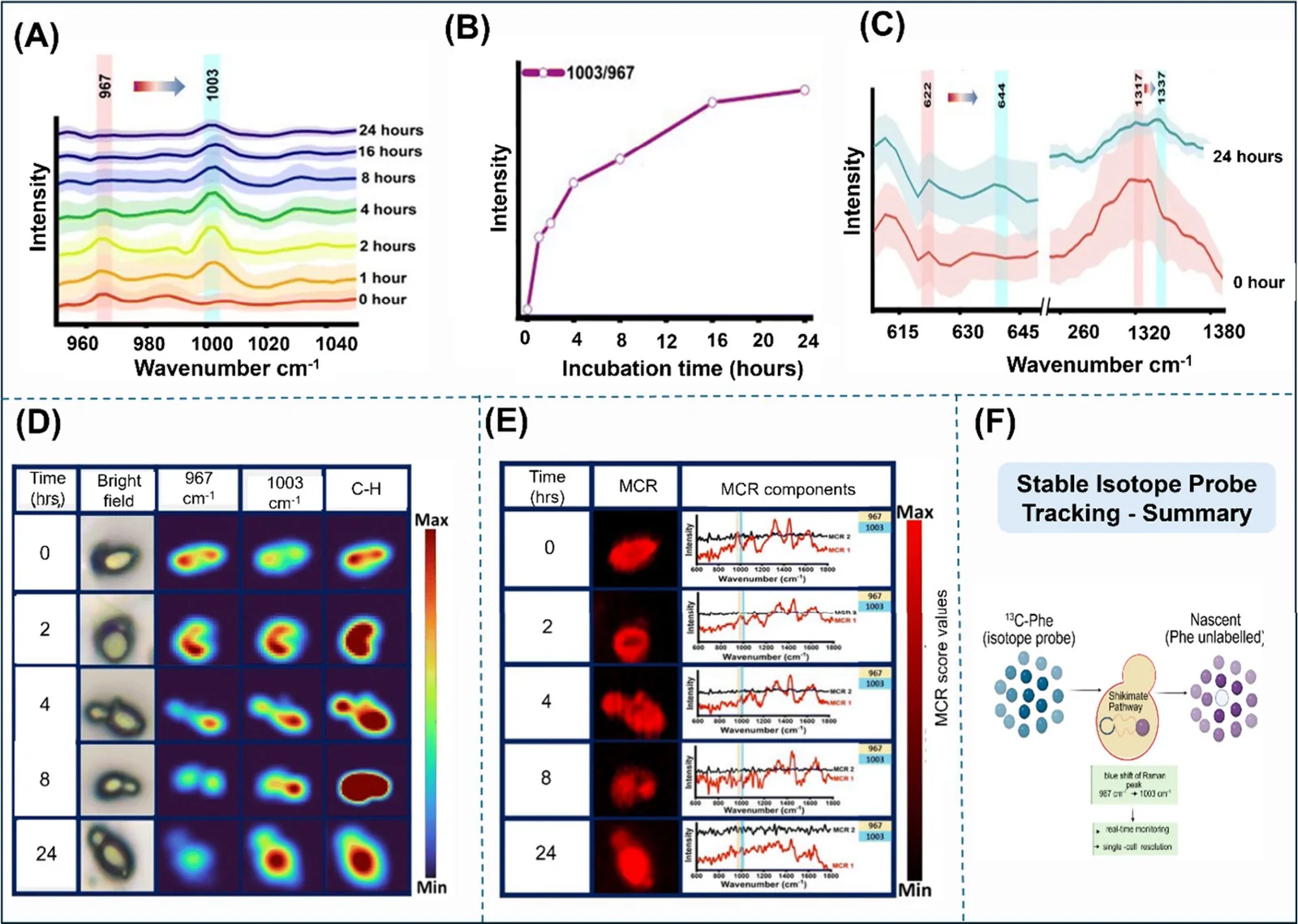
Raman stable isotope probing (SIP) enables real-time monitoring of shikimate pathway activity in single yeast cells. (A–C) Time-resolved Raman spectra showing isotope-induced shifts in phenylalanine, tyrosine, and tryptophan peaks and changes in phenylalanine turnover. (D, E) Single-cell Raman imaging and multivariate curve resolution (MCR) analysis tracking replacement of ^13^C-labeled phenylalanine (976 cm^–1^) by newly synthesized phenylalanine (1003 cm^–1^). (F) Schematic summarizing nondestructive, real-time metabolic tracking using Raman SIP. Panels A–F adapted with permission from ref 353 (Copyright 2024 Royal Society of Chemistry; Available under CC BY-NC 3.0 License).

## Biodistribution and Pharmacokinetics

*In vivo* Raman tracking has depicted that SERS nanoprobes remain chemically stable in zebrafish tissues, exhibiting minimal reporter degradation during early development.^354^ Nanoprobe retention in yolk sac, somite, and craniofacial regions confirms Raman labels maintain spectral integrity in diverse tissues.^355^ The persistence of distinct Raman signatures across multiple organs suggests stable probe-biomolecule interactions that do not disrupt developmental signaling or nascent vasculature.^356^ These biodistribution patterns demonstrate the capabilities of SERS nanoprobes to map developmental transport processes with high sensitivity.^354^ Lipid-coated Raman probes were shown to improve biocompatibility because lipid interfaces mimic natural membrane components and reduce inflammatory response.^357^ Studies on lipid-wrapped gold nanoparticles have demonstrated reduced macrophage uptake, suggesting that lipid coatings may prolong circulation and extend imaging windows *in vivo*.^357^ Additionally, lipid-nanoparticle hybrids exhibit enhanced colloidal stability in serum-like environments, supporting predictable pharmacokinetics during systemic administration.^358^ To model the probe clearance mechanism, it was shown in zebrafish that NPs are mainly cleared via hepatobiliary and pronephric pathways, resulting in time-dependent probe reduction.^359,360^ Clearance is also size-dependent with gold NPs larger than ∼40 nm in diameter exhibiting delayed clearance, emphasizing the necessity of size-optimized Raman probe design for safe *in vivo* application^361,362^ (Figure 15).

**Figure 15:**
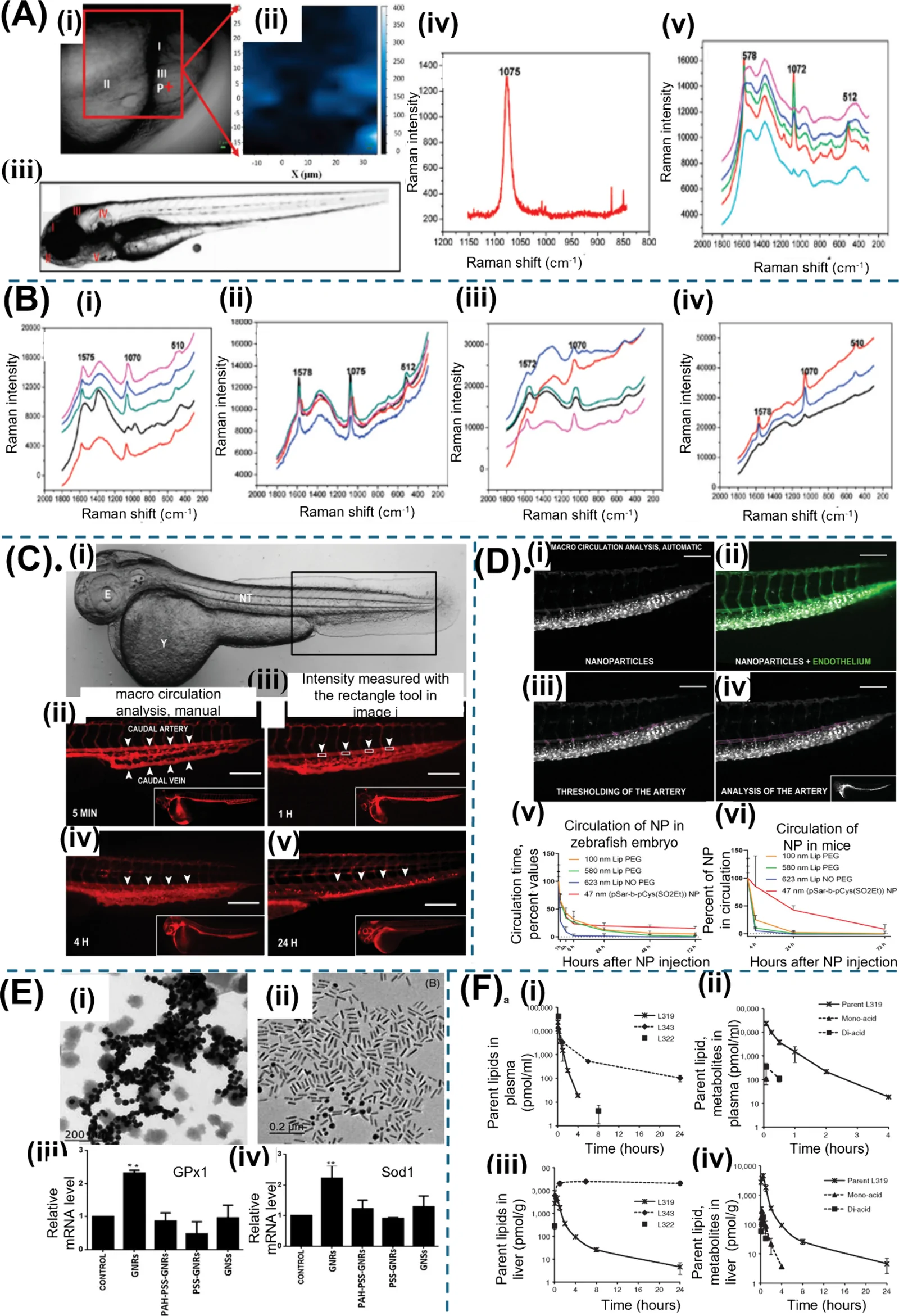
Representative applications of SERS and lipid nanoparticles (LNPs) in zebrafish and mouse models. (A) Raman mapping of 20–22 hpf zebrafish embryos showing SERS C–C band (1078 cm^–1^) distribution, anatomical structures, and corresponding tissue spectra. (B) SERS characterization of nanoparticles embedded in 96 hpf zebrafish from defined tissue regions. (C) Time-course imaging (5 min–24 h) of 100 nm fluorescent PEG-liposome circulation in 48 hpf zebrafish embryos with quantified fluorescence decay (scale bar: 200 μm). (D) Automated MACRO-based analysis of nanoparticle circulation in zebrafish and comparison with mice across multiple nanoparticle formulations (*N* ≥ 4). (E) TEM characterization of gold nanospheres and nanorods and associated oxidative stress responses (Gpx1a and Sod1 expression) in zebrafish embryos (6–72 hpf). (F) Plasma and liver pharmacokinetics of LNP lipids (L319, L343, and L322) and L319 metabolites following intravenous administration (0.3 mg/kg siRNA) in mice. Panel A adapted with permission from ref 354 (Copyright 2010 American Chemical Society). Panels B, C, and D reproduced from ref 356 (Copyright 2020 Wiley-VCH GmbH). Available under CC BY-NC 4.0 License. Panel E adapted with permission from ref 362 (Copyright 2018 John Wiley & Sons, Ltd.). Panel F reproduced from ref 367 (Copyright 2013 The American Society of Gene & Cell Therapy). Available under CC BY-NC-ND 3.0 License.

Other factors include surface charge, which influences NP retention, with positively charged NPs exhibiting longer accumulation than neutral or anionic counterparts^363,362^ (Figure 15). Shape-dependent toxicity has also been observed, with rod-shaped gold nanostructures inducing higher developmental toxicity than spherical counterparts.^364,365^ These insights emphasize the importance of controlling physicochemical properties when designing lipid Raman nanoprobes for metabolic studies. To enhance translational relevance, future lipid-Raman constructs should incorporate degradable lipid architectures to facilitate controlled breakdown and minimize long-term tissue accumulation.^366,357^ Such biodegradable lipid interfaces could provide predictable pharmacokinetic profiles compatible with repeated dosing or chronic metabolic monitoring.^367^ Additionally, incorporating receptor-targeting lipids may direct Raman-active probes toward specific tissues, enabling organ-selective metabolic imaging in live organisms.^368^

## Tumor and Metabolic Imaging Applications

Recent advances in vibrational imaging, particularly SRS imaging, have enabled label-free, chemically selective, and *in vivo* mapping lipids, proteins, and water in live animal and human tissues with high spatial and temporal resolution.^351^ Early studies have demonstrated SRS imaging in mice and human skin with subcellular resolution and physiological conditions. As a result, SRS has emerged as a powerful platform for chemical-specific *in vivo* imaging without requiring exogenous fluorescent labels or radioactive tracers.^351^ More recently, SRS was combined with stable isotope metabolic labeling to significantly extend its capabilities. The method, known as deuterium oxide probing stimulated Raman scattering (DO-SRS), involves the administration of deuterium oxide (D_2_O) or deuterated amino acids to metabolically label newly synthesized biomolecules. These labeled molecules incorporate carbon–deuterium (C–D) bonds, vibrations of which were typically detected by Raman shifts.^164^ The system was demonstrated to image *de novo* lipogenesis and protein biosynthesis in mice and Zebrafish.^313,369^ The study showed that newly synthesized protein signals (C–D) accumulated more strongly within tumor tissue compared to surrounding stroma, indicating metabolic heterogeneity within the tumor *in vivo*.^313^ These findings underscore that the power of DO-SRS to simultaneously visualize lipid and protein metabolism across spatially complex tissues, revealing metabolic rewiring associated with tumor growth or other pathologies.^164,370^

The methodology has been extended to other Raman-active lipid reporters such as deuterated fatty acids, alkyne-tagged lipids, or vibrationally engineered nanoassemblies. It was used to quantify lipid uptake, droplet nucleation, membrane remodeling, and lipid peroxidation within various disease models such as tumors, inflamed tissues, or metabolically dysregulated organs under hypoxic, acidic, and steatotic conditions.^361^ Raman-encoded lipid constructs were able to provide real-time, chemically orthogonal measurements of lipid flux with subcellular resolution, surpassing the limitations of fluorescence and radiotracer methods.^371,372^ Hence, the integration of Raman-addressable lipid reporters with *in vivo* SRS platforms defines a next-generation strategy for high-specificity metabolic imaging. This approach also enables multiplexed imaging across the pathological microenvironments.

Beyond metabolism, Raman-based techniques have also been applied for other functional imaging of therapy response. For example, in a mice tumor model, label-free Raman Spectroscopy was used to detect drug-induced apoptosis *in vivo* by observing spectral shifts in cellular components such as DNA, protein, lipid, and collagen bands. The study shows that apoptotic regions exhibited lower DNA Raman signal (*e*.*g*. at ∼ 785 cm^–1^) compared to normal tissue, providing a noninvasive readout of therapeutic efficacy.^373^ These results demonstrate that Raman-based imaging hold considerable promises for multiplexed metabolic and molecular imaging of tumors.

## Neurological Diseases

Neurological disorders affect millions worldwide and rank among the leading contributors to disability adjusted life years (DALYs).^374^ Raman-based vibrational imaging offers chemical specificity, label-free operations, and strong intrinsic multiplexing capacity, enabling direct interrogation of molecular pathology in complex brain tissues.^375^ In Alzheimer’s disease (AD), β-amyloid (Aβ) plaque deposition and lipid-droplet accumulation have been presented clinically. However, the mechanistic links among these processes remain incompletely understood.^376,268,377^ One theory suggest that early mitochondrial dysfunction including reduced cytochrome-c oxidase activity, elevated reactive oxygen species (ROS), and impaired energy metabolism might play a role in disease progression in AD.^378,379^

Neuroinflammation further amplifies oxidative stress and lipid imbalance in AD.^380,379,381–383^ Disrupted lipid metabolism has also been linked to lysosomal storage disorders and other neurodegenerative diseases.^384^ Lipids in the brain function not only as structural components but also as fast signaling molecules such as endocannabinoids, which regulate synaptic activity, development, and inflammation.^385^ These lipid messengers are shown to be synthesized on demand and are tightly regulated by metabolic enzymes.^386,387^ Chemical biology approaches, including bioorthogonal lipids, activity-based probes, photoreactive lipids, and genetically encoded sensors, have demonstrated real-time visualization of these lipid-metabolizing enzymes (DAGLα/β, MAGL, ABHD6/12, FAAH, NAPE-PLD, and NAAA).^386^ Although lab-based studies using lipid nanoparticles (LNPs)-conjugated nucleic acid therapies have shown promise for treating neurodegeneration, brain tumors, and stroke, their efficient delivery across the blood-brain barrier (BBB) remains a major bottleneck.^388^ To better evaluate and optimize brain delivery, high-throughput BBB trans-well screening platforms have been developed to assess both mRNA-LNP formulations^389,388^ (Figure 16). Recent studies have shown proof-of-principle gene editing in nonhuman primates and human patient-derived brain tissues.^390^

**Figure 16:**
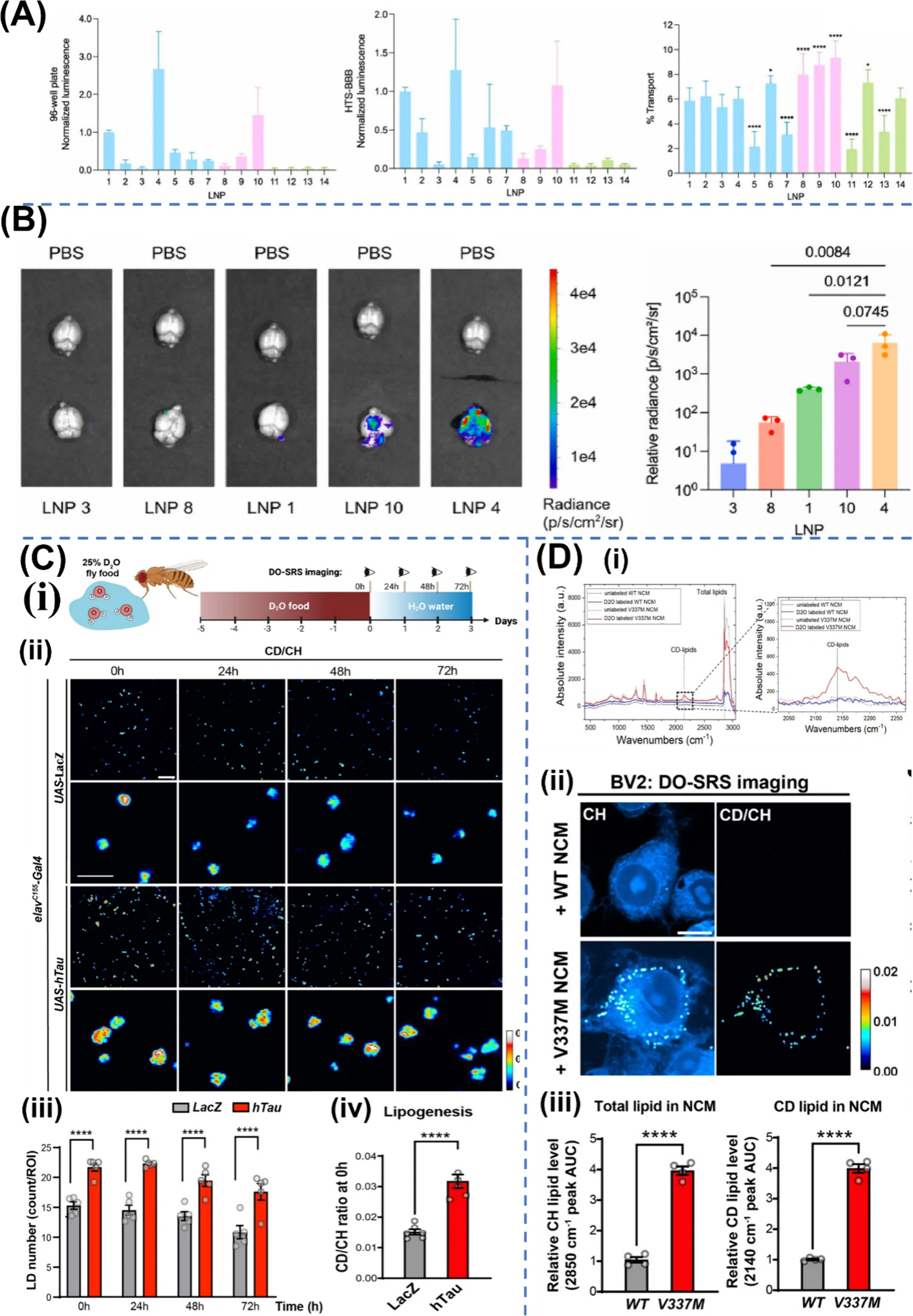
Representative applications of Raman imaging and lipid nanoparticles (LNPs) in neurological disease models. (A) High-throughput blood–brain barrier (HTS-BBB) screening identified LNP4 and LNP10 as effective brain-delivery candidates, with LNP10 exhibiting superior transport efficiency *in vitro* and LNP4 demonstrating the highest brain-targeting specificity *in vivo*. (B) DO-SRS imaging of lipid droplet dynamics in hTau *Drosophila* brains following D_2_O labeling and starvation, revealing altered lipid droplet abundance and *de novo* lipogenesis in wild-type versus hTau brains. (C) (i–iv) DO-SRS analysis of V337 M iPSC neuron–microglia interactions showing deuterium-labeled lipid transfer, lipid droplet accumulation, pro-inflammatory responses, and impaired phagocytosis in BV2 cells, supported by quantitative C–D (2140 cm^–1^) and C–H (2850 cm^–1^) imaging and Raman spectra. (D) V337 M iPSC neurons transfer newly synthesized lipids to microglia, inducing LD accumulation, inflammation, and impaired phagocytosis. (i) DO-SRS detected D_2_O-labeled lipids only in V337M-NCM-treated BV2 cells­(72h). Scale bar, 10 μm. (ii) Total and D-labeled lipids quantified from the 2850 and 2140 cm^–1^ peaks. (iii) A unique 2140 cm^–1^ Raman peak confirms release of newly synthesized D-labeled lipids from V337 M neurons. Panel A adapted with permissions from ref 11 (Copyright 2023 Annual Reviews, Available under a Creative Commons Attribution 4.0 International License). Panels B–D adapted with permission from ref 377 (Copyright 2024 Elsevier Inc.).

Raman studies successfully probed protein synthesis. In this work, misfolded Aβ exhibited a ∼10 cm^–1^ blue shift in the amide I Raman band compared with normal proteins, allowing label-free identification of plaques using multicolor SRS. These results were validated using immunohistochemical imaging.^391–394^ Spontaneous Raman microspectroscopy of post-mortem human AD brain tissue was used to detect abnormal lipid species, including saturated cholesteryl esters, β-carotene, and magnetite colocalized with Aβ plaques, with strong spatial gradients from plaque cores to peripheries.^395,396^ In another study, researchers have used carbon nanotube-based lipid sensors to investigate the sphingomyelin mechanism in the brain.^384^ Other advancements include hyperspectral Raman and multimodal Raman. Hyperspectral Raman imaging was used to detect plaque maturation via the β-sheet amide I Raman bands.^355^ For the multimodal Raman system, SRS, two-photon autofluorescence, and SHG were integrated.^355,397^

## Infectious Diseases, Immune Response, and Inflammation

Raman spectroscopy has been used for monitoring inflammation and immune activation with chemical specificity and multiplexing capability.^398^ The study used human microvascular endothelial cells and induced inflammatory stimuli and hyperglycemic conditions. Raman imaging revealed increased lipid production, high lipid unsaturation, and altered mitochondrial activity, reflecting inflammation-driven metabolic reprogramming.^398^ These findings provide proof-of-concept demonstration for the Raman system’s utility to study inflammation-associated metabolic shifts. Host–pathogen interactions often trigger extensive remodeling of host lipid metabolism, membrane composition, and subcellular lipid organization.^399^ Raman-based lipid imaging provides a chemically specific, label-free method to monitor such remodeling at subcellular resolution.^400^ For instance, Raman imaging was used in human macrophages exposed to oxidized low-density lipoprotein (oxLDL) to track lipid trafficking using deuterated lipids.^401,402^

Raman spectroscopy has also been applied directly to probe pathogen themselves.^403^ In this study, *in vivo Mycobacterium tuberculosis* (Mtb) model was used to investigate bacterial composition. It found bacteria population with ‘lipid-rich (dormant) and ‘lipid poor’ (non-dormant) states.^403^ The bacteria with lipid-rich states were correlated with relapse infection. This research highlights the potential of Raman-based lipid imaging to discriminate microbial metabolic states while simultaneously interrogating host lipid responses.^404^

Immune cell activation typically leads to metabolic reprogramming, with increased lipid synthesis, lipid droplet formation, membrane remodeling, and altered lipid saturation.^404^ Although an imperfect classification, recent Raman studies have distinguished naive, pro-inflammatory (M1) and anti-inflammatory (M2) macrophage phenotypes solely by lipid composition, without using fluorescent labels or antibodies.^405^ In another study, using THP-1 monocyte cells with LPS and IFN-γ, Raman analysis detected significant changes in protein, phospholipid, nucleic acid, and nonmembrane lipid signatures, reflecting activation-induced biochemical remodeling.^405^ These observations support the use of endogenous Raman lipid signals as label-free reporters of immune phenotype and functional state. Inflammation could also promote the formation of lipid mediators (eicosanoids, prostaglandins) and lipid droplets (LD) within immune and endothelial cells.^406^ Raman microscopy has been used to resolve LD heterogeneity in hepatocytes from nonalcoholic steatohepatitis (NASH) models, revealing subcellular differences in lipid chemical composition.^407^ Additionally, recent work has used the natural Raman-active dye astaxanthin (AXT), which colocalizes with cellular lipids in endothelial cells. This labeled Raman approach enabled spatiotemporal imaging of lipid-rich compartments under inflammatory conditions and highlights its potential as a universal lipid probe.^408^

A key strength of Raman imaging is its ability to simultaneously differentiate various cellular components in a single measurement.^405^ Hence, combining lipid-based Raman probes with other modalities such as fluorescence, coherent Raman scattering, or fiber-based Raman endoscopy could provide multimodal insights into immune cell identity and metabolic state. It would also provide spatial context within infected tissues.^409^ While lipid-based Raman nanoprobes for infection and inflammation are still exploratory, future efforts should focus on optimizing biocompatibility, improving specificity, and improving signal-to-noise performance in preclinical and clinical tissue.^409^ Applying established immunology and biomaterials evaluation frameworks to infection and inflammation models is crucial for translation.^410^ Ultimately, minimally invasive *in vivo* Raman approaches could allow longitudinal monitoring of immune responses, pathogen metabolism, and therapeutic efficacy, opening new avenues for diagnostics and mechanistic immunopathology research.^411^

## Metabolic Disorders

Raman-based metabolic imaging, as mentioned above, is a strong, noninvasive method for probing lipid storage dynamics and subcellular lipid heterogeneity, offering direct molecular insights into metabolic dysregulation.^400,412^ In the context of drug-induced liver injury (DILI), a recent study has introduced an endogenous peroxynitrite (ONOO^—^) responsive bioorthogonal SERS nanoprobe for metabolic liver injury imaging.^412,273^ By utilizing a selective α-ketoamide-peroxynitrite reaction, this probe enabled ratiometric detection in the Raman-silent spectral region and real-time single-cell tracking of ONOO^—^ induction and inhibition. Importantly, this platform mapped acetaminophen-induced nitrosative stress in HepG2 cells and DILI mouse livers, revealing mechanisms of oxidative and nitrosative hepatoxicity.^412^

In another study of *Caenorhabditis elegans* model, SERS imaging probed dynamic lipid mobilization and heterogeneity among lipid droplet populations across intestinal tissues for real-time metabolic tracking.^361^ These studies established SRS-derived vibrational signatures as reliable markers of lipid flux during physiological perturbations, laying a foundation for translational metabolic imaging.^361,400^ Extending Raman approaches to mammalian models, spontaneous Raman microscopy has detected hepatic lipid accumulation in early nonalcoholic fatty liver disease (NAFLD) before histological changes could be detected.^413^ In mouse models of nonalcoholic steatohepatitis (NASH), Raman imaging enabled spatial mapping of LD within hepatocytes, while spectral analysis quantified variations in lipid saturation and cholesterol content.^407^ Notably, changes in acyl-chain unsaturation and cholesterol distribution correlated with disease severity, highlighting molecular-level remodeling during NAFLD-NASH progression.^407^

Dual-band hyperspectral SRS was applied to biological samples. In this study, hyperspectral SRS was used to image liver tissues from patients with NAFL or NASH. Using a machine-learning-based analysis approach, the study was able to perform accurate classification and quantification of triglycerides, saturated and unsaturated cholesteryl esters, and free cholesterol, spatially.^339^ A spontaneous Raman system was also demonstrated to probe metabolic dysfunction in white adipose tissue in response to a high-fat diet in mice.^414^ The system was also utilized to probe fatty acid changes in the tumor microenvironment in TNBC.^415^ Complementing imaging, Raman spectroscopy of biofluids is advancing as a minimally invasive diagnostic tool. A recent clinical study applied label-free SERS to filter serum samples of patients with type II diabetes mellitus and healthy controls.^416^ The study has demonstrated that diabetic and control sera have distinct spectral fingerprints, including phospholipid-linked, protein-linked, and small-molecule peaks, enabling accurate classification of diabetic versus nondiabetic serum samples.^416^ Hence, the integration of Raman microscopy with lipid-specific nanoprobes and SERS-based diagnostics could emerge as a clinically relevant tool for studying metabolic disorders.^63^

## Translational Bottlenecks for Raman-Based Metabolic Imaging

Although Raman-based imaging provides high chemical specificity and subcellular-scale biochemical information, its clinical translation faces a different set of barriers than those of established metabolic imaging modalities such as PET, SPECT, MRI, and CT. The main translational bottleneck is not a single factor (Table 5) such as sensitivity; rather, it depends on whether the Raman approach is label-free, nanoparticle-enhanced, or isotope/probe-assisted.

**Table 5:** Comparison of Different Preclinical Modalities with Established Clinical Modalities

Modality	Current clinical maturity	Typical spatial scale	Metabolic readout	Sensitivity bottleneck	Calibration/quantification bottleneck	Pharmacokinetics/biodistribution bottleneck	Toxicity	Regulatory complexity	Instrumentation access	Translational bottleneck
Label-free spontaneous Raman	Low–moderate; mostly *ex vivo* or intraoperative studies	∼0.5–1 μm laterally; shallow optical depth	Full-spectrum biochemical fingerprint: lipids, proteins, nucleic acids, metabolites	Moderate–high: spontaneous Raman scattering is intrinsically weak	High: spectra depend on preprocessing, baseline correction, tissue heterogeneity, and model transfer	Low: no exogenous probe	Low: label-free and nondestructive	Moderate: device/software validation rather than drug approval	Moderate: Raman microscopes exist, but clinical Raman systems are not routine	Speed, depth, calibration, and workflow integration
SERS/Raman nanotags	Low; mostly preclinical	Subcellular to tissue-scale depending on delivery and optics	Targeted detection or nanoparticle tracking	Low: very sensitive because plasmonic enhancement can be large	Very high: signal depends on hot spots, aggregation, local environment, and nanoparticle distribution	Very high: nanoparticle delivery, clearance, RES uptake, and tumor penetration are major barriers	High: gold/silver/plasmonic materials require safety and clearance evaluation	Very high: combined device + nanoparticle/drug-like probe pathway	Low–moderate	Pharmacokinetics, toxicity, calibration, and regulatory complexity
Label-free SRS/CARS	Low–moderate; emerging intraoperative and tissue-imaging use	∼0.3–1 μm laterally; depth typically hundreds of μm in tissue	Fast lipid/protein contrast; limited-band chemical imaging	Moderate: better than spontaneous Raman but lower than nuclear imaging	Moderate: SRS is more linear than CARS; CARS has nonresonant background	Low: no exogenous probe	Low	Moderate: device validation	Low: ultrafast laser systems remain specialized	Instrumentation complexity, depth, and clinical workflow
Isotope/bioorthogonal SRS	Low; mostly research	∼0.3–1 μm laterally; tissue depth similar to SRS	Metabolic flux: lipid synthesis, protein synthesis, nutrient uptake	Moderate–low: Raman-silent region improves contrast	Moderate–high: requires control of isotope dose, incubation time, and metabolic incorporation	Moderate: depends on isotope/probe delivery	Moderate: isotope or analog probes can perturb metabolism depending on dose and structure	High: administration of metabolic probes adds regulatory burden	Low	Probe administration, metabolic perturbation, and instrumentation
FDG-PET/PET-CT	Very high; routine clinical metabolic imaging	Clinical resolution commonly several mm	Glucose uptake/tracer-specific metabolism	Low: extremely sensitive radiotracer detection	Moderate: standardized uptake value, partial-volume correction, scanner calibration	Moderate: tracer PK is well characterized for approved tracers	Moderate: ionizing radiation; pharmacologic mass usually very low	High but established: approved PET drugs and IND pathways exist	High in tertiary centers	Cost, radiation, tracer availability, and limited molecular specificity
SPECT/SPECT-CT	Very high; routine clinical functional imaging	Lower resolution than PET, typically mm–cm scale	Perfusion, receptor, bone turnover, thyroid, cardiac function depending on tracer	Low–moderate: less sensitive and lower resolution than PET	Moderate	Moderate: tracer PK well characterized for approved agents	Moderate: ionizing radiation	High but established	High	Lower sensitivity/resolution than PET, radiation, and tracer specificity
MRI/MRS	Very high for MRI; moderate for clinical MRS	∼mm for MRI; larger voxels for MRS	Anatomy, perfusion, diffusion, metabolites by MRS	High for MRS: intrinsically lower sensitivity than PET	Moderate–high: metabolite quantification needs field homogeneity and spectral fitting	Low–moderate: often no tracer; contrast agents may be used	Low–moderate; no ionizing radiation, but gadolinium safety issues in selected patients	Established for MRI; variable for new probes	Very high	Sensitivity for low-abundance metabolites and spectral resolution
Hyperpolarized^**13**^C MRI	Emerging clinical translation	mm scale	Real-time metabolic flux, especially pyruvate-to-lactate conversion	Moderate: hyperpolarization boosts ^13^C signal by >10,000-fold but signal decays rapidly	High: timing, polarization level, kinetic modeling, and acquisition protocols are critical	Moderate: injected hyperpolarized substrate	Moderate: exogenous metabolic substrate administered	High: drug-device workflow	Low–moderate; requires polarizer and specialized MRI workflow	Short signal lifetime, workflow complexity, and standardization
CT/perfusion CT	Very high	Submm to mm	Primarily anatomy; perfusion/vascular surrogate, not direct metabolism	Low for anatomy; poor molecular sensitivity	Moderate for perfusion models	Moderate: iodinated contrast PK matters	Moderate: radiation and iodinated contrast risks	Established	Very high	Not chemically specific for metabolism; radiation/contrast burden

The comparison highlights that Raman-based metabolic imaging should not be judged by the same translational criteria as PET or SPECT.^417^ These modalities are clinically mature, because they provide whole-body, highly sensitive tracer detection through standardized radiopharmaceutical workflows. FDG-PET, for example, is widely used for oncologic metabolic imaging, however, its spatial resolution is typically within the millimeter range,^418^ and its dominant biological readout is glucose analog uptake rather than full biochemical composition.^419^ PET radiotracers also follow a well-established regulatory pathway;^420^ FDA guidance explicitly describes investigational new drug (IND) processes for investigational PET drugs,^421^ and clinical PET drugs may be used after new drug application (NDA) or abbreviated new drug application (ANDA) approval.^422^ Therefore, for PET, the major translational challenges are not sensitivity or clinical access but radiation exposure, tracer availability, cost, and limited molecular specificity beyond the chosen tracer.

By contrast, the central bottleneck for label-free spontaneous Raman imaging is not toxicity or pharmacokinetics as no exogenous probe is required. Instead, its major barriers are weak signals, slow acquisition, limited optical penetration, and calibration across instruments, tissues, and preprocessing pipelines.^312^ These limitations matter clinically, because a diagnostic platform must provide robust and reproducible results across operators, instruments, tissue handling conditions, and patient cohorts. However, spontaneous Raman has a unique translational advantage: it provides a full-spectrum biochemical fingerprint of tissue chemistry, typically including fingerprint and high-wavenumber regions rather than a single-tracer readout. Therefore, spontaneous Raman is best positioned as a chemically specific, label-free approach for *ex vivo* pathology, intraoperative margin assessment, and metabolic phenotyping, rather than as a whole-body metabolic imaging modality such as PET.^313^

For SRS and CARS, the bottleneck shifts from signal generation to clinical instrumentation and depth. Coherent Raman methods overcome the speed limitations of spontaneous Raman and enable rapid imaging of live cells and fresh tissues, but they typically require ultrafast lasers, complex optical alignment, and specialized detection. SRS is generally more quantitatively attractive than CARS because the SRS signal is directly proportional to molecular concentration under appropriate conditions,^312^ whereas CARS suffers from a nonresonant background that complicates chemical specificity and quantification.^423^ Therefore, SRS/CARS are promising for intraoperative or fresh-tissue imaging, but broad translation requires compact, robust, and clinically deployable systems.

For SERS and lipid/Raman nanoparticle probes, the bottlenecks are fundamentally different. Although sensitivity is often excellent, clinical translation is limited by NP pharmacokinetics, biodistribution, clearance, long-term retention, batch-to-batch reproducibility, toxicity, and regulatory complexity. This is especially important for plasmonic gold or silver particles, where Raman intensity depends on local electromagnetic hotspots, aggregation state, reporter orientation, and tissue delivery.^316^ Thus, SERS probes are compelling for multiplexed local detection, sentinel applications, and *ex vivo* assays, but they face a higher translational burden than label-free Raman because the probe itself becomes a regulated material requiring safety, manufacturing, and biodistribution evaluation.^315^

Isotope- and bioorthogonal-probe SRS occupy an intermediate translational position. It offers more direct metabolic information than label-free SRS because isotope incorporation or Raman-silent tags can report lipid synthesis, protein synthesis, or substrate utilization. However, the administration of D_2_O, ^13^C substrates, alkynes, nitriles, or other reporters introduces additional considerations regarding the dosage, timing, metabolic perturbation, and regulatory classification. This makes isotope-SRS powerful for mechanistic and preclinical metabolic imaging but less immediately translatable than label-free Raman or established PET tracers.^46,424^

Compared with Raman technologies, MRI and hyperpolarized ^13^C MRI are clinically more mature in terms of infrastructure but less sensitive for low-abundance molecules. Hyperpolarized ^13^C MRI directly addresses metabolism by imaging real-time conversion of injected substrates such as [1-^13^C]­pyruvate to lactate. Hyperpolarization can enhance ^13^C MRI signal by more than 10,000-fold, but the signal decays rapidly, requiring tightly coordinated substrate preparation, injection, and imaging.^425^ Thus, its bottleneck is not chemical specificity but workflow complexity, standardization, and limited availability of polarizers and trained teams.

Finally, CT and perfusion CT are highly mature clinically but are not true molecular metabolic imaging modalities. CT provides excellent anatomical resolution and can quantify perfusion with an iodinated contrast, but it does not provide direct biochemical specificity for lipids, proteins, metabolites, or metabolic flux. Its main translational strengths are speed, availability, and standardization; its limitations are ionizing radiation, contrast-related risks, and lack of molecular metabolic information.^426^

In summary, Raman-based technologies are most realistic for near-term clinical translation in settings where their high chemical specificity and microscopic resolution add value beyond PET, MRI, or CT—particularly *ex vivo* pathology, intraoperative tissue assessment, endoscopic probes, margin detection, and localized metabolic phenotyping.

## Future Perspective

In the future, optimized spectral unmixing and AI-driven approaches will enable highly multiplexed Raman probes. The design of bioorthogonal and enzyme-responsive tags will be able to probe metabolic states both *in vitro* as well as *in vivo*.^427–429,121^ Nanoprobe designs could take inspiration from cell-based immunotherapies, such as CAR-T and tumor-infiltrating lymphocyte (TIL) strategies, to enhance tissue penetration, target specificity, and microenvironment modulation.^430^ Similarly, biodegradable lipid nanocarriers could preserve Raman sensitivity while enabling favorable renal clearance, thereby reducing the risk of toxicity. The development of alternative conjugation materials, such as doped organic semiconductors or transient metallic constructs, could further minimize long-term toxicity.^431–434,377^ Additionally, standardized metrics and good manufacturing practices (GMP)-based synthesis are essential to advance these probes toward clinical translation.^435–437^

## Data Availability

No data has been produced in this study.

## Vocabulary

Bioorthogonal Raman Reporter: A small, chemically inert vibrational tag (such as alkyne, nitrile, or carbon-deuterium bond) that absorbs and scatters light exclusively within the cellular silent region (1800–2700 cm^–1^) without disrupting native cellular biochemistry.
Stable Isotope Probing (Raman-SIP): A method combining Raman microspectroscopy with stable isotope-labeled precursors (like D_2_O or ^13^C-glucose) to track metabolic incorporation and turnover dynamics at single-cell resolution.
Nanostructured Lipid Carrier (NLC): A second-generation lipid nanoparticle composed of solid and liquid lipid blends that form a disordered matrix, offering superior physical stability and high encapsulation efficiency for hydrophobic payloads and optical reporters.
Coherent Anti-Stokes Raman spectroscopy (CARS): A four-wave mixing nonlinear optical process utilizing two laser beams to coherently drive molecular vibrations, generating high-frequency anti-Stokes signals ideal for rapid-rich structural imaging.
Surface-Enhanced Raman Scattering (SERS): A sensitive analytical technique that amplifies Raman scattering signals by orders of magnitude through electromagnetic “hot spots” generated by localized surface plasmon resonance on noble metal nanostructures.
Stimulated Raman Scattering (SRS): A nonlinear optical imaging technique that uses synchronized pump and Stokes laser beams to stimulate molecular vibrations, providing rapid, quantitative, and background-free chemical imaging.

## Abbreviations

4-MBA: 4-Mercaptobenzoic acid
AD: Alzheimer’s disease
AF: Autofluorescence
AI: Artificial intelligence
AIE: Aggregation-induced enhancement
AMPK: AMP-activated protein kinase
APOE4: Apolipoprotein E4
AuNPs: Gold nanoparticles
BBB: Blood–brain barrier
BRRs: Bioorthogonal Raman reporters
CARS: Coherent anti-Stokes Raman spectroscopy
CDDM: Clearance-Degradation Decoupling Model
CNNs: Convolutional neural networks
CRS: Coherent Raman spectroscopy
CSRS: Coherent Stokes Raman scattering
D_2_O: Deuterium oxide
D_2_O: Heavy water (deuterium oxide)
DALYs: Disability adjusted life years
DCF: *In situ* drift correction factor
DILI: Drug-induced liver injury
DLS: Dynamic light scattering
EARPs: Enzyme-activated Raman probes
ECRC: Effective cellular Raman concentration
EF: Enhancement factors
ESR: Executable spectroscopy records
FLI: Fluorescence imaging
FLIM: Fluorescence lifetime imaging microscopy
FS: Fluorescence spectroscopy
GNRs: Gold nanorods
GNSs: Gold nanospheres
iPSC: Induced pluripotent stem cells
LD: Lipid droplets
LNPs: Lipid nanoparticles
LOD: Limit of detection
M1: Pro-inflammatory
M2: Anti-inflammatory
MARS: Manhattan Raman scattering
MMP: Matrix metalloproteinase
MPE: Maximum permissible exposure
MRI: Magnetic resonance imaging
MS: Mass spectrometry
Mtb: *Mycobacterium tuberculosis*
NAFLD: Non-alcoholic fatty liver disease
NASH: Non-alcoholic steatohepatitis
NIR: Near-Infrared
NLCs: Nanostructured lipid carriers
NMF: Non-negative matrix factorization
NPA: Niemann-Pick type A
NpCN1: 1,8-Naphthalimides
OPF: Optical performance fingerprint
OPO: Optical parametric oscillator
ORQI: Organelle-Resolved Quantification Index
oxLDL: Oxidized low-density lipoprotein
PA: Photoacoustic
PAH: Polyallamine hydrochloride
PEG: PEGylated
PET: Positron emission tomography
PMT: Photomultiplier tube
PPM: Physiological phantom matching
PSS: Polystyrene
PVAT: Perivascular adipose tissue
QbD: Quality by design
RES: Reticuloendothelial system
RQCI: Raman quantification confidence interval
RSDI: Reporter Surface Density Index
SENN: Spectral Extraction Neural Network
SERS: Surfaced enhanced raman spectroscopy
SHG: Second harmonic generation
SLNs: Solid-lipid nanoparticles
SNR: Signal-to-noise ratio
SORS: Spatially offset Raman spectroscopy
SRS: Stimulated Raman scattering
TNBC: Triple-negative breast cancer
TPF: Two-photon fluorescence
TPM: Two-photon microscopy
WMR: Wavelength-modulated Raman
